# Weiyan Tongluo Granules attenuate gastric intestinal metaplasia through PPARγ/NF-κB/CDX2 signaling pathway

**DOI:** 10.1186/s13020-026-01350-y

**Published:** 2026-03-04

**Authors:** Yimin Liang, Shijie Su, Ting Jin, Junyu Mou, Hui Wu, Ning Yan, Xiang Li, Simeng Yao, Yi Wen, Fengbin Liu, Peiwu Li

**Affiliations:** 1 The First Clinical College, Guangzhou University of Chinese Medicine, Guangzhou, 510405 China; 2 Lingnan Medical Research Center, Guangzhou University of Chinese Medicine, Guangzhou, 510405 China; 3 Postdoctoral Research Station, Guangzhou University of Chinese Medicine, Guangzhou, 510405 China; 4 Guangdong Clinical Research Academy of Chinese Medicine, Guangzhou, 510405 China; 5 The Second Clinical College, Guangzhou University of Chinese Medicine, Guangzhou, 510405 China; 6 The Eighth Clinical Medical College of Guangzhou University of Chinese Medicine, Foshan, 528000 China; 7 Department of Hepatobiliary Diseases, The First Affiliated Hospital of Guangzhou University of Chinese Medicine, Guangzhou, 510405 China; 8 Lingnan Institute of Spleen and Stomach Diseases, The First Affiliated Hospital of Guangzhou University of Chinese Medicine, Guangzhou, 510405 China; 9 Baiyun Hospital of the First Affiliated Hospital of Guangzhou University of Chinese Medicine, Guangzhou, 510470 China; 10 State Key Laboratory of Traditional Chinese Medicine Syndrome, The First Affiliated Hospital of Guangzhou University of Chinese Medicine, Guangzhou, 510405 China

**Keywords:** Weiyan Tongluo Granules, Gastric intestinal metaplasia, Transcriptomics, PPARγ/NF-κB/CDX2 signaling pathway, Apoptosis, Traditional Chinese medicine

## Abstract

**Background:**

Gastric intestinal metaplasia (GIM) is a precancerous condition characterized by the replacement of gastric mucosa with intestinal-like epithelium, posing a considerable risk for gastric cancer with no effective cure available. Weiyan Tongluo Granules (WYTLG), a traditional Chinese medicine (TCM) formulation, has demonstrated clinical potential in managing GIM, though its mechanisms remain elusive. This study sought to systematically investigate the therapeutic effects of WYTLG on GIM and its molecular mechanism.

**Methods:**

An integrative strategy combining in vivo, in vitro, and in silico methodologies was conducted. We identified the main compounds of WYTLG and then established GIM models in rats and cells through induction using N-methyl-N’-nitro-N-nitrosoguanidine and deoxycholic acid to evaluate its pharmacological effects. Transcriptomic analysis was performed on gastric tissues to fully elucidate the underlying mechanisms. In addition, molecular docking analysis was employed to predict the interactions between key compounds of WYTLG and potential targets. The mechanistic findings were further investigated and validated through cellular-level experiments.

**Results:**

A total of 29 components were identified in WYTLG. Treatment with WYTLG dose-dependently improved gastric injury and GIM in rat models, accompanied by restoration of gastric function, attenuation of pathological alterations, and regulation of GIM-related biomarkers. WYTLG treatment significantly alleviated inflammatory responses and enhanced resistance to apoptosis in gastric glands. Consistent with the in vivo findings, WYTLG-containing serum exerted comparable anti-GIM effects in vitro. Transcriptomic analysis highlighted the PPARγ/NF-κB signaling pathway as a key mechanism underlying the effects of WYTLG. These finding was further corroborated by intervention studies using GW9662, a PPARγ antagonist, TNF-α, an NF-κB activator, and si-PPARγ, all of which largely attenuated the therapeutic effects. Furthermore, molecular docking analysis demonstrated favorable binding affinities between active WYTLG components and key targets within this pathway.

**Conclusion:**

WYTLG attenuates the progression of GIM through modulation​ of the PPARγ/NF-κB/CDX2 axis, providing a prospective strategy for preventing gastric malignant transformation.

**Graphical Abstract:**

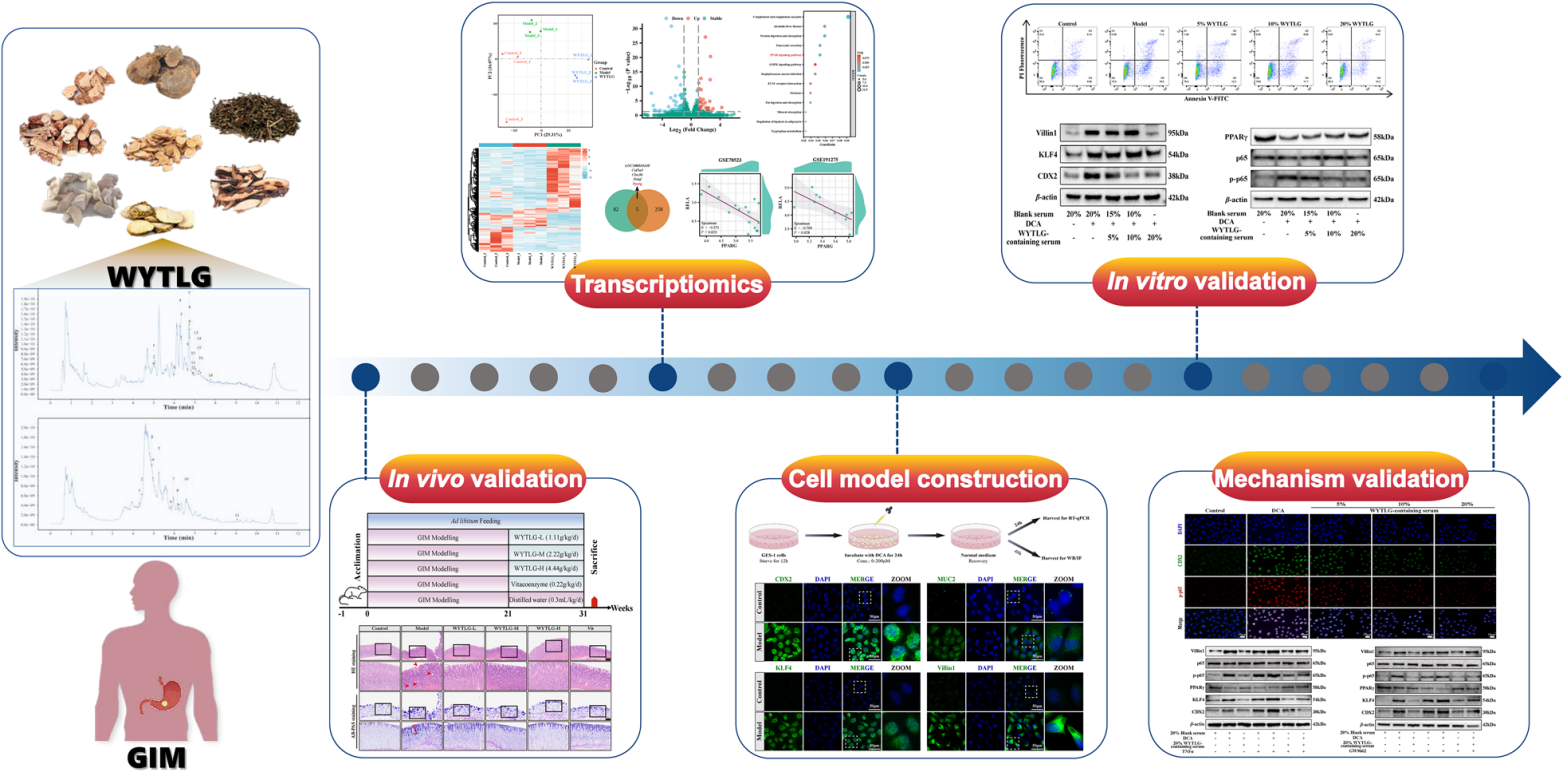

**Supplementary Information:**

The online version contains supplementary material available at 10.1186/s13020-026-01350-y.

## Introduction

Gastric intestinal metaplasia (GIM) refers to the replacement of gastric mucosa with intestinal-like epithelium [1]. Correa first synthesized observations of gastritis, atrophy, and metaplasia into a stepwise progression of gastric cancer (GC) development [2], in which GIM plays a pivotal intermediate role. Among GIM patients, the cumulative progression rates to intestinal-type GC at 3, 5, and 10 years were 0.4%, 1.1%, and 1.6%, respectively [3]. In East Asia, where GC is common, the prevalence of GIM is 21% [4]. However, the pathogenesis of GIM remains incompletely understood, resulting in insufficient risk stratification and limited therapeutic options. Hence, GIM was viewed as the “point of no return” by researchers [5]. This underscores the urgent need to seek safe and effective therapeutics for GIM.

An emerging body of evidence highlights the unique therapeutic advantages of traditional Chinese medicine (TCM) in managing GIM [6, 7]. Compared to conventional therapies, TCM has been shown to enhance clinical efficacy while reducing adverse effects. Through its characteristic multi-component, multi-target and multi-pathway modes of action, TCM aims not only to reverse gastric precancerous lesions (GPL) but also to restore holistic health [8–10]. Weiyan Tongluo Granules (WYTLG) is a herbal formulation under a Chinese patent application (No. CN119925543A) ​developed by Professor Fengbin Liu at the First Affiliated Hospital of Guangzhou University of Chinese Medicine. Derived from the classical *Si-Jun-Zi-Tang* and optimized through modern clinical practice, WYTLG has been widely applied in the clinical management of GIM. Preliminary studies have demonstrated WYTLG’s clinical efficacy in relieving upper abdominal discomfort, with marked improvements in key symptoms including bloating, pain, and nausea [11]. These therapeutic effects may be, at least in part, associated with modulation of the NF-κB signaling pathway [12]. The formulation consists of eight medicinal components: *Astragalus membranaceus* (Fisch.) Bge.var.mongholicus (Bge.) Hsiao [Huangqi] (15 g), *Ficus simplicissima* Lour. [Wuzhimaotao] (20 g), *Atractylodes macrocephala* Koidz. [Baizhu] (10 g), *Scutellaria barbata* D.Don [Banzhilian] (20 g), *Curcuma phaeocaulis* Val. [Ezhu] (10 g), *Sparganium stoloniferum* Buch.-Ham. [Sanleng] (10 g), *Margaritifera Concha* [Zhenzhumu] (20 g), and *Citrus aurantium* L. [Zhiqiao] (10 g). According to TCM theory, GIM is primarily caused by spleen-stomach Qi deficiency with resultant Qi stagnation, blood stasis, and damp-heat accumulation [13]. WYTLG is designed to invigorate the spleen, replenish Qi, clear heat and promote diuresis, activate blood circulation, and resolve stasis, thereby restoring the functional balance of the middle energizer. Modern medical research indicates that the nonspecific symptoms of GIM, are largely attributed to gastric mucosal thinning, atrophy, and glandular metaplasia, pathological features that align with the TCM syndrome patterns of Qi deficiency and blood stasis [6]. However, the main compounds and precise mechanisms of WYTLG are not yet completely understood.

In this study, we first conducted a quality control measure of WYTLG compounds. The GIM rat model was then used to evaluate its efficacy in vivo. Subsequently, we performed transcriptomic profiling on gastric tissues following WYTLG treatment coupled with bioinformatics analysis to explore its potential mechanisms. Moreover, a GIM cell model was established to validate the efficacy and mechanisms of WYTLG in vitro.

## Methods and materials

### Identification of the main compounds in WYTLG

The identification of the main compounds in WYTLG was performed using an ultra-high-performance liquid chromatography (UHPLC) coupled with untargeted metabolomics analysis. Three biological replicates were analyzed. Briefly, 500 μL of extraction solvent (methanol: acetonitrile: water = 2:2:1, v/v/v) was added to dilute the WYTLG powder, then centrifuged to collect the supernatant. Chromatographic separation was conducted using a Vanquish UHPLC system (Thermo Fisher Scientific, USA) equipped with a Phenomenex Kinetex C18 column (2.1 mm × 100 mm, 2.6 μm particle size). Mobile phase A comprised water with 0.01% acetic acid, while mobile phase B was a 1:1 (v/v) mixture of isopropanol and acetonitrile. The gradient elution program was as follows: 0–1 min, 99% A; 1–8 min, 99% to 1% A; 8–9 min, 1% A; returned to 99% A at 9.1 min and maintained until 12 min. The sample tray temperature was maintained at 4 °C, with a flow rate of 0.3 mL/min and an injection volume of 2 μL. The system was integrated with an Orbitrap Exploris 120 mass spectrometer (Thermo Fisher Scientific, USA) to acquire MS/MS spectra. Data were converted into mzXML format for subsequent peak extraction, identification, integration, and retention time alignment. The identified compounds were annotated by matching MS/MS spectra against the Biotree TCM (v1.0) and BT-HERB (v1.0) databases, followed by manual verification based on the Chinese Pharmacopoeia and relevant published literature. The detailed parameter settings are provided in Table S1.

### Animal experiment

Male Sprague–Dawley rats (130–150 g) were obtained from Guangdong Medical Laboratory Animal Center, acclimated for one week and used at six weeks of age. No more than five rats were housed in a cage with wood shavings under controlled conditions (20–26 °C, 40–70% humidity, 12-h light/dark cycle). The rats were allowed ad libitum chow and water unless otherwise indicated. All procedures followed the guidelines of the Institutional Animal Care and Use Committee of Guangzhou University of Chinese Medicine (Approval No. 20230315014, date: March 15, 2023). Humane endpoints were established to minimize animal suffering, including termination without compromising experimental outcomes, or if animals exhibited severe distress (e.g., rapid weight loss, reduced food intake, inability to eat/drink, hypothermia, or severe vomiting/diarrhea).

Seventy-two rats with comparable body weights were stratified into control (*n* = 12) and model groups (*n* = 60). The control group received distilled water and SPF-grade feed ad libitum. The model group underwent a multifactorial GIM induction protocol simulating human pathogenesis that involves excessive nitrite intake, bile reflux, and irregular dietary patterns, all of which are known risk factors for gastric mucosal injury and metaplasia [14, 15]. Specifically, on feeding days, the model group freely consumed N-Methyl-N’-nitro-N-nitrosoguanidine (MNNG; Macklin, China) at a concentration of 170 μg/mL in light-proof bottles [16–18]; on fasting days (twice weekly), feed was withdrawn, and only distilled water was provided, followed by oral gavage of a 20 mmol/L deoxycholic acid (DCA; Sigma-Aldrich, USA) solution before feeding resumed [19–21]. The control group received equivalent water gavage on matching days as the model group. Commencing at week 15, biweekly gastric sampling enabled histopathological monitoring until GIM confirmation. The diagnostic criteria for GIM were based on the ‘Chinese consensus on chronic gastritis (2017, Shanghai) [13].’

Following the establishment of the GIM rat model, the animals ​were randomized into 5 groups: model group, low-dose WYTLG (WYTLG-L, 1.11 g/kg/day), medium-dose WYTLG (WYTLG-M, 2.22 g/kg/day), high-dose WYTLG (WYTLG-H, 4.44 g/kg/day), and a Vitacoenzyme (Vit) group (0.22 g/kg/day). All treatments were administered by gavage. The dosing regimen was determined using the human-to-rat body surface area conversion ratio (0.018) according to​ *Experimental Methodology of Pharmacology* by Xu Shuyun [22], with the low, medium, and high doses corresponding to 1-, 2-, and fourfold of the human clinical equivalent dose, respectively. ​During the intervention period, all groups maintained the established modeling conditions. The control group underwent identical gavage procedures with distilled water without modeling. Each rat received a gavage volume of 0.3 mL/kg/d for 10 consecutive weeks. The survival status and body weight of the rats were dynamically monitored. Terminal tissue collection was performed​ under intraperitoneal anesthesia with 1% sodium pentobarbital, and gastric tissues were harvested for gross morphological examination and calculation of the stomach index (stomach weight-to-body weight ratio).For serum preparation, twenty rats were divided into the drug-containing serum group and the blank serum group. The drug group received WYTLG (4.44 g/kg/d, equivalent to fourfold of the human clinical dose) by gavage, while the blank group received distilled water, twice daily for 3 consecutive days. One hour following the last administration, blood samples were collected from the abdominal aorta under anesthesia, then centrifuged at 3,500 rpm for 15 min, heat-inactivated at 56 °C for 30 min, and cryopreserved at −80 °C until further use.

### Histopathological staining and analysis

Fresh gastric samples were fixed in 4% paraformaldehyde (PFA), dehydrated through an ethanol gradient, embedded in paraffin and sectioned at 3–4 µm thickness. Tissue sections underwent deparaffinization with xylene, followed by rehydration through a graded ethanol series. Staining procedures, including hematoxylin and eosin (H&E) and Alcian blue/Periodic acid-Schiff (AB/PAS) (Servicebio, China), were performed following the manufacturer’s protocols. After dehydration and xylene clearing, sections were mounted using neutral balsam. Pathological evaluation was conducted using a slide scanner (3DHISTECH, Hungary). To quantify goblet cell positivity, five random fields per section were analyzed using ImageJ software. A detection threshold was applied to identify AB/PAS-positive (blue or purple) areas. The percentage of positive cells was calculated as (positive cells/total cells) × 100% for each field, and the mean value of five fields was used for statistical analysis.

### Enzyme-linked immunosorbent assay (ELISA)

Serum or cell supernatant levels of pepsinogen Ⅰ (PG Ⅰ), pepsinogen Ⅱ (PG Ⅱ), gastrin-17 (GAS-17), and inflammatory cytokines (IL-1β, IL-6, TNF-α, and IL-10) were measured using commercial ELISA kits (Meimian, China) as instructed. Absorbance was read at 450 nm with a microplate reader (Thermo Fisher Scientific, USA). The pepsinogen ratio (PGR) was calculated as PG Ⅰ/PG Ⅱ.

### Reverse transcription quantitative polymerase chain reaction (RT-qPCR)

Total RNA was extracted from gastric tissues or cells using the RNA Purification Kit (EZBioscience, USA) according to the manufacturer’s instructions. RNA concentration and purity were measured using a NanoDrop 2000 spectrophotometer (Thermo Fisher Scientific, USA). Subsequently, cDNA was synthesized from RNA through reverse transcription using the Color Reverse Transcription Kit (EZBioscience, USA). The mRNA levels were quantified utilizing the 2 × SYBR Green Premix qPCR Kit on a CFX384 PCR System (Bio-Rad, USA). *β*-actin was used as a housekeeping gene. Primer information is detailed in Supplementary Table S2.

### Immunohistochemistry (IHC)

Gastric tissue sections were deparaffinized and rehydrated according to previously established protocols. Antigen retrieval was conducted by microwave heating of sections in citrate buffer (pH 6.0)​. After cooling to room temperature (RT), sections were treated with 3% H_2_O_2_ for 20 min to block endogenous peroxidase, followed by 10% goat serum blocking at 37 °C for 1 h. Subsequently, they were incubated overnight at 4 °C with the primary antibodies: anti-CDX2 (Proteintech, #82,659–1-RR, Wuhan, China, 1:2000) or anti-MUC2 (Proteintech, #27675–1-AP, Wuhan, China, 1:2000). On the next day, sections underwent a 1-h incubation at RT with HRP-conjugated Goat anti-Rabbit IgG (H + L) (Servicebio, #GB23303, Wuhan, China, 1:200). Freshly prepared DAB chromogen was applied for incubation​ of sections while monitoring ​using​ a brightfield microscope (Leica, Germany). Sections were counterstained with hematoxylin before mounting.

### TdT-mediated dUTP nick-end labeling (TUNEL) assay

Apoptosis in gastric tissues was assessed using the TUNEL Assay Kit (Servicebio, China) as instructed. After standard deparaffinization/rehydration as previously described, proteinase K was applied to the sections at 37 °C for 20 min to enhance permeability. Following equilibration with equilibration buffer at RT for 15 min, sections were incubated with a TdT mixture (recombinant TdT enzyme, TMR-5-dUTP labeling mix, and equilibration buffer; 2:5:50, v/v/v) at 37 °C for 1 h in the dark. Finally, slides were mounted with DAPI-containing medium (Biosharp, China) and imaged by fluorescence microscopy (Olympus, Japan) under appropriate fluorescence channels.

### Transcriptomic and bioinformatics analysis

Gastric tissue RNA from the control group, the model group, and the WYTLG-H group (dose selection was based on therapeutic efficacy results) was extracted and quantified as previously described. RNA integrity was then assessed using an Agilent 2100 Bioanalyzer (Agilent Technologies, USA). RNA sequencing was performed on the Illumina NovaSeq 6000 (Illumina, USA), with gene expression quantified as fragments per kilobase per million mapped reads (FPKM). Differential expression analysis was conducted using DESeq2 (v1.20.0), applying a significance threshold of adjusted *p*-value < 0.05 and |log2 fold change|> 1. Differentially expressed genes (DEGs) were enriched for GO and KEGG pathways using the clusterProfiler (3.8.1) package in R software (v4.2.1).

To validate the clinical relevance of key DEGs, transcriptomic datasets ​of human GIM mucosal tissues​ were downloaded from the Gene Expression Omnibus (GEO) database (https://www.ncbi.nlm.nih.gov/geo/) under accession numbers ​GSE78523 and GSE191275. The correlation of key DEGs in microarray expression data was analyzed and visualized using the ​ggplot2 package (v3.4.4)​​.

### Cell modeling, grouping and intervention

The immortalized human gastric epithelial cell line GES-1 and gastric adenocarcinoma cell line AGS were maintained in RPMI-1640 medium (Gibco, USA) supplemented with 10% fetal bovine serum (FBS; Gibco, USA) and 1% penicillin/streptomycin (Gibco, USA) at 37 °C in an incubator with 5% CO2 (Memmert, Germany), and harvested with 0.25% trypsin–EDTA (NCM Biotech, China). All cells were stored in a liquid nitrogen tank.

For GIM modeling, both GES-1 and AGS cells were seeded in 6-well plates (1 × 10^5^ cells/well) and cultured until attachment. Following serum starvation in RPMI-1640 for 12 h, cells were exposed to DCA for 24 h to establish GIM cells. After DCA removal, cells were restored in complete medium or subjected to drug-containing serum intervention at different concentrations, and then harvested at two time points: 24 h for RT-qPCR analysis and 48 h for Western blotting or immunofluorescence (IF) assays.

### Cell viability assay

Cell viability was assessed by cell counting kit-8 (CCK-8; Biosharp, China) according to manufacturers’ protocols. In brief, cells were seeded in 96-well plates (1 × 10^4^ cells/well) and cultured to 70–80% confluency. The experimental groups were then treated with varying concentrations of DCA, drug-containing serum, TNF-α (31–45) (MedChemexpress, USA), or GW9662 (MedChemexpress, USA). Following another 24 h incubation, CCK-8 solution was added and incubated for 1 h at 37 °C in the dark. Absorbance was read at 450 nm with a microplate reader (Thermo Fisher Scientific, USA).

### Immunofluorescence

Cells were seeded in 24-well plates (5 × 10^4^ cells/well) containing cell slides until attachment. Following 12-h serum starvation, cells were exposed to 200 μM DCA for 24 h, followed by treatment with varying concentrations of drug-containing serum. After rinsing, the cells were fixed with 4% PFA at RT for 30 min. For intracellular targets, cells were permeabilized with 1–5% Triton (Biosharp, China) for 15 min. 10% goat serum was applied to block non-specific binding at 37 °C for 30 min. Primary antibodies, including anti-Villin1 (Proteintech, #16,488–1-AP, Wuhan, China, 1:250), anti-KLF4 (Proteintech, #11,880–1-AP, Wuhan, China, 1:200), anti-CDX2 (Immunoway, #YM3057, Jiangsu, China, 1:200), and anti-MUC2 (Proteintech, #27,675–1-AP, Wuhan, China, 1:400), were applied, and plates were sealed with parafilm and incubated overnight at 4 °C. The next day, cells were incubated in the dark for 1 h with Alexa Fluor 488-conjugated Goat anti-Rabbit IgG (H + L) (Thermo Fisher Scientific, #A-11008, USA, 4 μg/mL) or Alexa Fluor 594-conjugated Goat anti-Mouse IgG (H + L) (Thermo Fisher Scientific, #A-11005, USA, 5 μg/mL) cross-adsorbed secondary antibody. Coverslips were mounted with DAPI-containing antifade medium and imaged using appropriate fluorescence channels.

### Flow cytometry analysis

Annexin V-FITC Apoptosis Detection Kit (Beyotime, China) was utilized to assess cell apoptosis, following the provided instructions. Drug-treated GIM cells from six-well plates were trypsinized and centrifuged to obtain a cell pellet. The pellet was then resuspended in PBS, adjusting the cell density to 1 × 10^5^ cells per sample. Subsequently, Annexin V-FITC binding buffer, Annexin V-FITC, and PI solution were added and mixed. After incubation at RT for 15 min in the dark. Samples were kept on ice and analyzed within 1 h using a flow cytometer (BD Biosciences, USA).

### Western blotting

Treated cells or tissues were lysed in RIPA buffer containing phosphatase and protease inhibitors (Thermo Fisher Scientific, USA) on ice for 30 min, then centrifuged to obtain the supernatant. Protein concentration was determined using BCA Protein Assay Kits (Thermo Fisher Scientific, USA). Proteins were separated by 10–15% SDS-PAGE gradient gels and transferred to PVDF membranes, which were then blocked with 5% BSA or skim milk for 1 h at RT. Primary antibodies were diluted as follows: anti-Villin1 (Proteintech, #16,488–1-AP, Wuhan, China, 1:2000), anti-KLF4 (Proteintech, #11,880–1-AP, Wuhan, China, 1:5000), anti-CDX2 (Proteintech, #82,659–1-RR, Wuhan, China, 1:2500), anti-SOX2 (Proteintech, #11,064–1-AP, Wuhan, China, 1:1000), anti-cytochrome c (Abmart, #T55734F, Shanghai, China, 1:1000), anti-Bax (Abmart, #T40051F, Shanghai, China, 1:1000), anti-Bcl-2 (Abmart, #T40056, Shanghai, China, 1:1000), anti-cleaved caspase-3 (CST, #9661S, Danvers, MA, USA, 1:1000), anti-PPARγ (Proteintech, #16,643–1-AP, Wuhan, China, 1:2500), anti-p65 (CST, #8242S, Danvers, MA, USA,1:1000), anti-Phospho-p65 (CST, #3033S, Danvers, MA, USA,1:1000), and anti-*β*-actin (Immunoway, #YT0099, Jiangsu, China, 1:5000), and incubated overnight at 4 °C. The next day, membranes were incubated with HRP-conjugated Goat anti-Rabbit IgG (NCM Biotech, #P8002, Jiangsu, China, 1:2000) for 1 h at RT. ECL reagents were applied for chemiluminescent detection, and signals were captured with a Bio-Rad gel imaging system (Bio-Rad, USA). *β*-actin served as the loading control for normalization and band intensities were quantified using ImageJ software.

### siRNA Transfection

siRNA targeting PPARγ and negative control siRNA were commercially obtained from GenePharma (Shanghai, China). The sequences of si-NC, si-PPARγ #1, and si-PPARγ #2 are listed in **Table S3**. GES-1 cells were seeded into 6-well plates at 3 × 10^5^ cells per well and transfected with si-NC or si-PPARγ using Lipofectamine 2000 (Thermo Fisher Scientific, USA) according to the manufacturer’s instructions. After 6 h, the medium was replaced with complete medium containing 10% FBS, and cells were cultured for 48–72 h before subsequent experiments.

### Molecular docking

The three-dimensional structures of the ligands were retrieved from the PubChem database (https://pubchem.ncbi.nlm.nih.gov/) and the crystal structures of the target proteins were obtained from the RCSB Protein Data Bank (https://www.rcsb.org/). Before docking, all ligand and receptor structures were prepared with AutoDock Tools (ADT; v1.5.6), which included the addition of hydrogen atoms, assignment of charges, and definition of rotatable bonds. Molecular docking was then performed using AutoDock Vina. The docking grid was centered on the protein’s binding site with dimensions sufficient to encompass the entire cavity. A conformational search was carried out, and the resulting poses were ranked based on binding affinity, expressed as the calculated binding free energy (ΔG, kcal/mol). Docking poses with ΔG < –1.2 kcal/mol were considered biologically plausible. The affinity results were visualized using a clustering heatmap.

### Statistical analysis

Data were analyzed using GraphPad Prism (v9.5.0, GraphPad Software, San Diego, CA, USA). Data normality was assessed using the Shapiro–Wilk test. Normally distributed data are expressed as mean ± SEM and analyzed by one-way ANOVA with Bonferroni or Dunnett’s post hoc tests for multiple comparisons. Non-normally distributed data are presented as median (interquartile range) and analyzed using the Kruskal–Wallis test with Bonferroni-adjusted comparisons. Student’s *t* test was used for two-group comparison. The correlation between PPARγ and RELA expression was examined using Spearman’s correlation analysis. A *p*-value < 0.05 indicates a significant difference.

## Results

### WYTLG improves the general condition and gastric injury of GIM rats

**Fig. 1:**
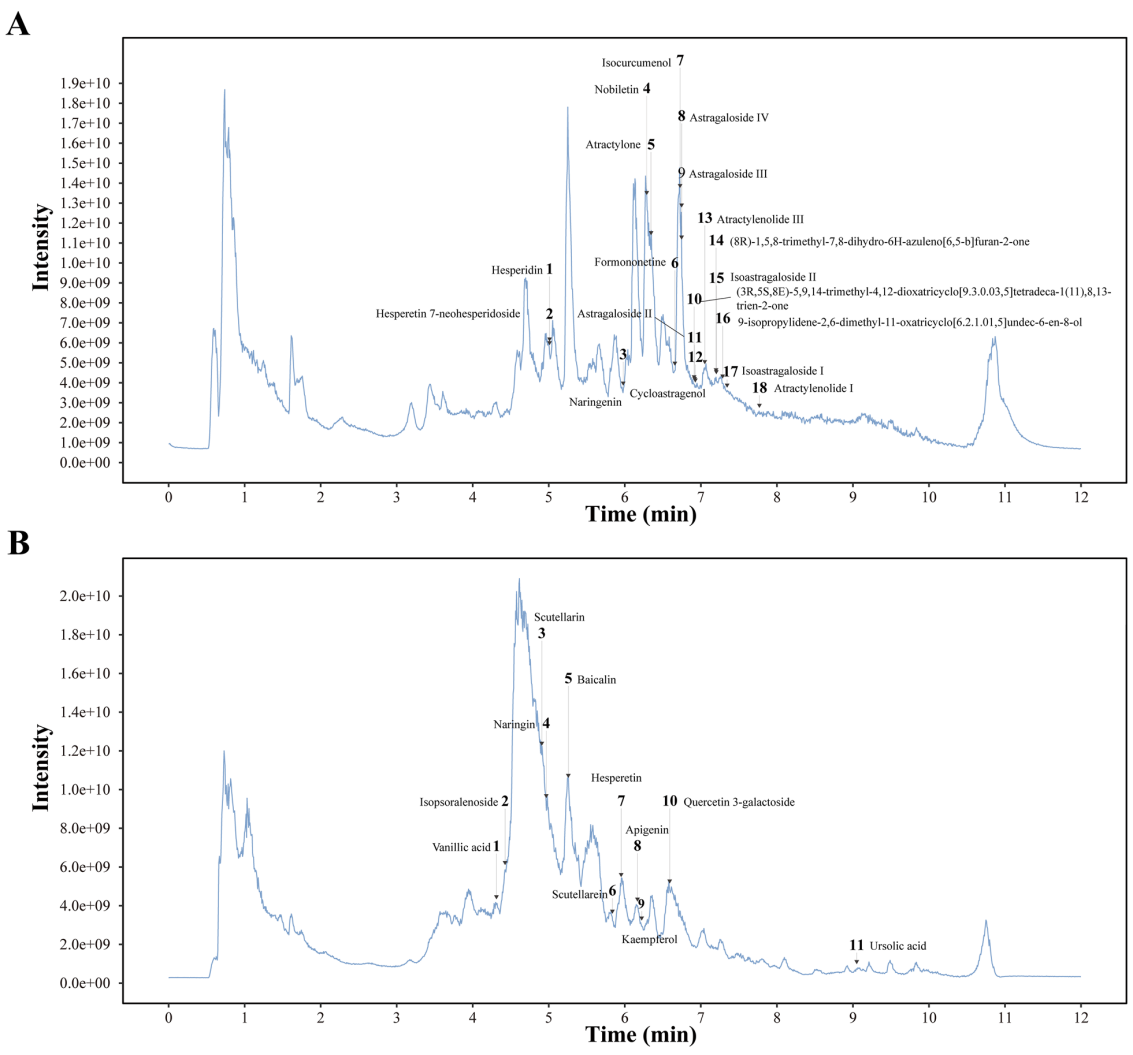
Total ion chromatogram of WYTLG over time**. A** Positive ion mode chromatogram. **B** Negative ion mode chromatogram

A comprehensive chemical profiling of the WYTLG formula was performed using UPLC-MS/MS analysis. Total ion chromatograms (TIC) were obtained in both positive and negative ion modes (Fig. 1). A total of 29 main compounds were identified, including 12 flavonoids, 7 sesquiterpenes, 7 triterpenes, 3 phenolic derivatives, with the source of each compound is detailed in Supplementary Table S4.

**Fig. 2:**
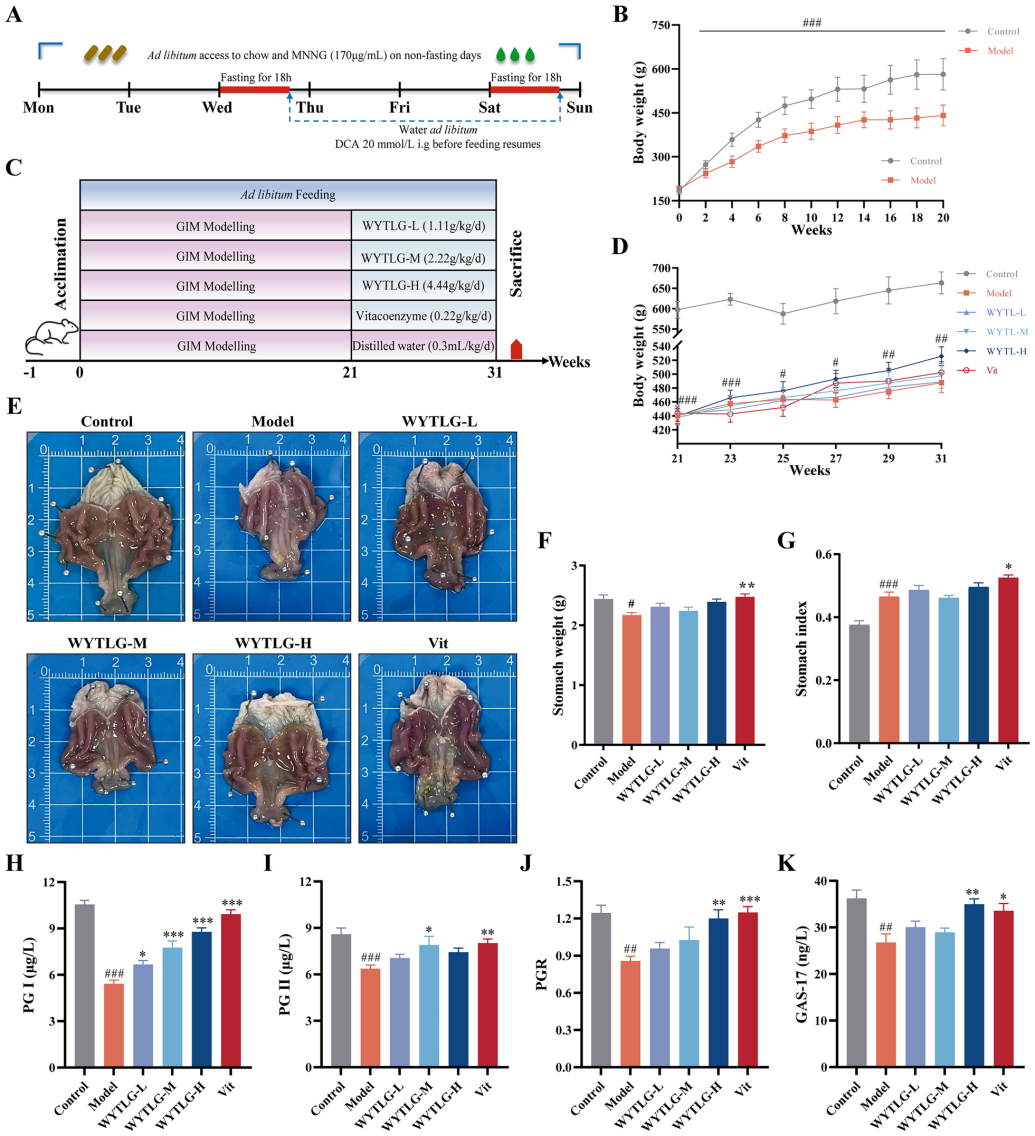
The therapeutic effects of WYTLG on general condition and gastric injury in GIM rats. **A** Schematic diagram of GIM modeling procedure.** B** Body weight changes during the modeling period. **C** Experimental grouping and treatment protocol. **D** Body weight changes during the treatment period. **E** Representative macroscopic images of gastric morphology (scale bar = 1 cm). **F** Statistical graph of stomach weight. **G** Statistical graph of stomach index. **H–K** Serum levels of gastric function markers. (*n* = 8). ^#^*p* < 0.05, ^##^*p* < 0.01, ^###^*p* < 0.001 *vs.* control group; ^*^p < 0.05, ^**^p < 0.01, ^***^p < 0.001 *vs.* model group

In this study, an in vivo GIM model was established to evaluate the therapeutic effects of WYTLG on GIM-associated symptoms (Fig. 2A). The model group showed significantly delayed weight gain compared to the control group, which was partially reversed by WYTLG (at varying doses) and Vit treatment (Fig. 2B-D). Macroscopic gastric examination revealed that the model group displayed reduced gastric size, dull mucosa, decreased and stiff rugae, swelling, significantly lower stomach weight, and elevated stomach index. These pathological changes were markedly alleviated following WYTLG and Vit intervention (Fig. 2E-G). The serological analysis demonstrated that PG Ⅰ, PG Ⅱ, PGR, and GAS-17 levels were significantly downregulated in the GIM model group (*p* < 0.01, *p* < 0.001), whereas WYTLG and Vit treatment partially restored these indicators (Fig. 2H-K). Collectively, these results indicate that WYTLG effectively improves the general condition and gastric dysfunction in GIM rats.

### WYTLG alleviates intestinal metaplasia lesions in GIM rats

**Fig. 3:**
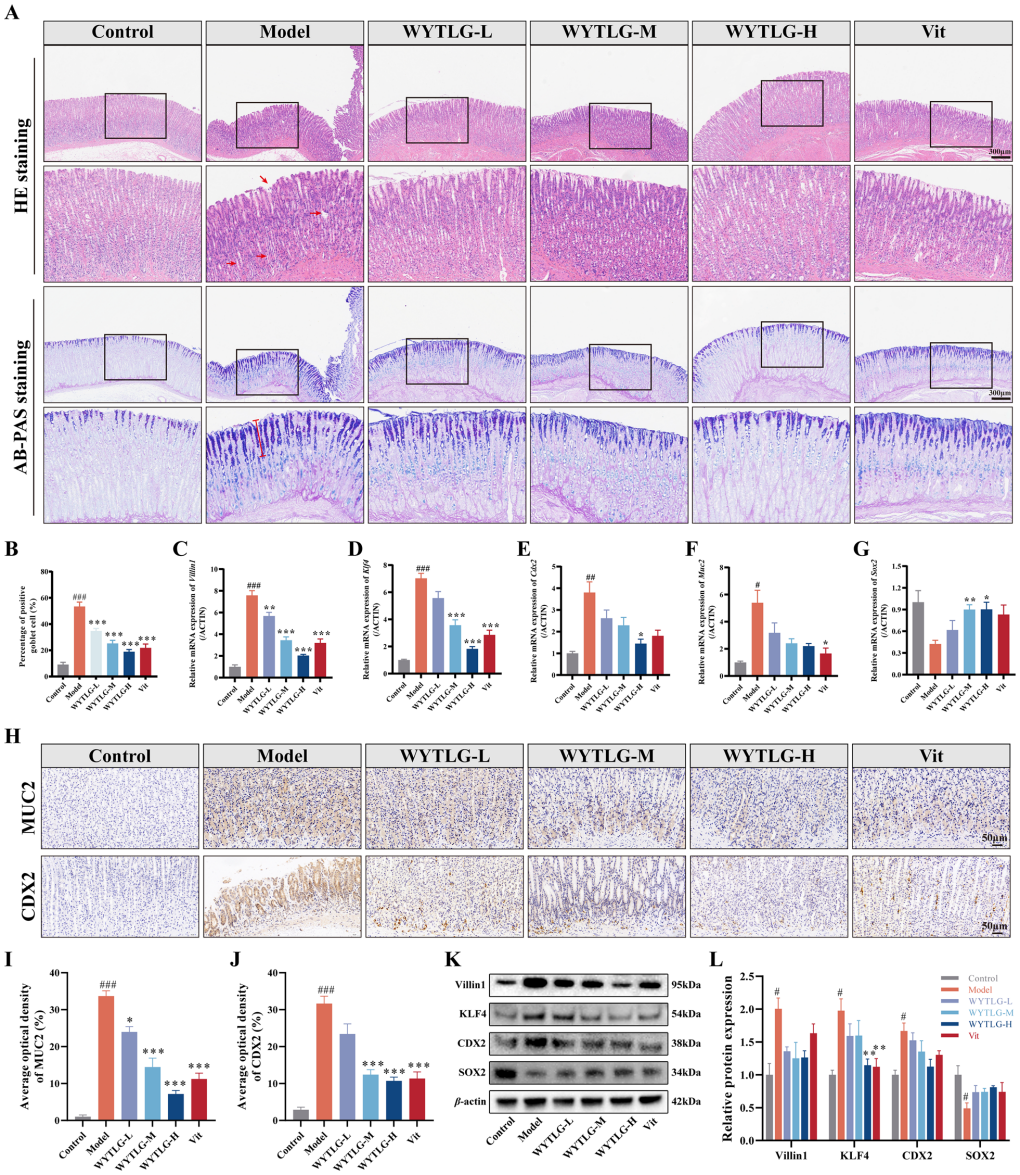
WYTLG alleviates lesions of intestinal metaplasia in GIM rats.** A** Representative images of H&E and AB/PAS staining (scale bar = 300 μm).** B** Goblet cell positivity (%)​. **C-G** Relative mRNA expression levels of *Villin1*, *Klf4*, *Cdx2*, *Muc2*, and *Sox2*. **H-J** Representative images of IHC staining and semi-quantitative analysis for MUC2 and CDX2 (scale bar = 50 μm). **K, L** Western blot analysis of Villin1, KLF4, CDX2, and SOX2 protein expression. (*n* = 3). ^#^*p* < 0.05, ^##^*p* < 0.01, ^###^*p* < 0.001 *vs.* control group; ^*^p < 0.05, ^**^p < 0.01, ^***^p < 0.001 *vs.* model group

To further evaluate the pathological and molecular changes of WYTLG on GIM at the microscopic level, gastric tissues were subjected to pathological staining and investigation of intestinal markers. As shown in Fig. 3A, the control group had intact glands, with arranged epithelial cells and no inflammatory cell infiltration. In contrast, the model group showed glandular atrophy, enlarged and disorganized glandular spacing, thinning of the mucosal layer, and inflammatory cell infiltration in the stroma, accompanied by Alcian blue-positive mucin secretion in goblet cells. Compared with the model group, WYTLG treatment dose-dependently reduced the proportion of goblet cells (*p* < 0.001), while the Vit group also showed a significant decrease (*p* < 0.001) (Fig. 3B).

Subsequently, we examined the mRNA (Fig. 3C-G) and protein (Fig. 3H-L) expression levels of key intestinal markers (Villin1, KLF4, CDX2, and MUC2) and the gastric marker SOX2 to further investigate the molecular alterations in GIM. In the model group, intestinal marker expression was significantly upregulated (*p* < 0.05), whereas SOX2 was downregulated (*p* < 0.05). WYTLG treatment dose-dependently reduced the expression of intestinal markers and restored SOX2 levels, with the most pronounced regulatory effects observed in the WYTLG-H group. In addition, the Vit group also showed significant modulation of these markers (*p* < 0.05). These results indicated that WYTLG has the potential to reverse GIM and curb malignant transformation.

### WYTLG modulates inflammatory responses and apoptosis in GIM rats

**Fig. 4:**
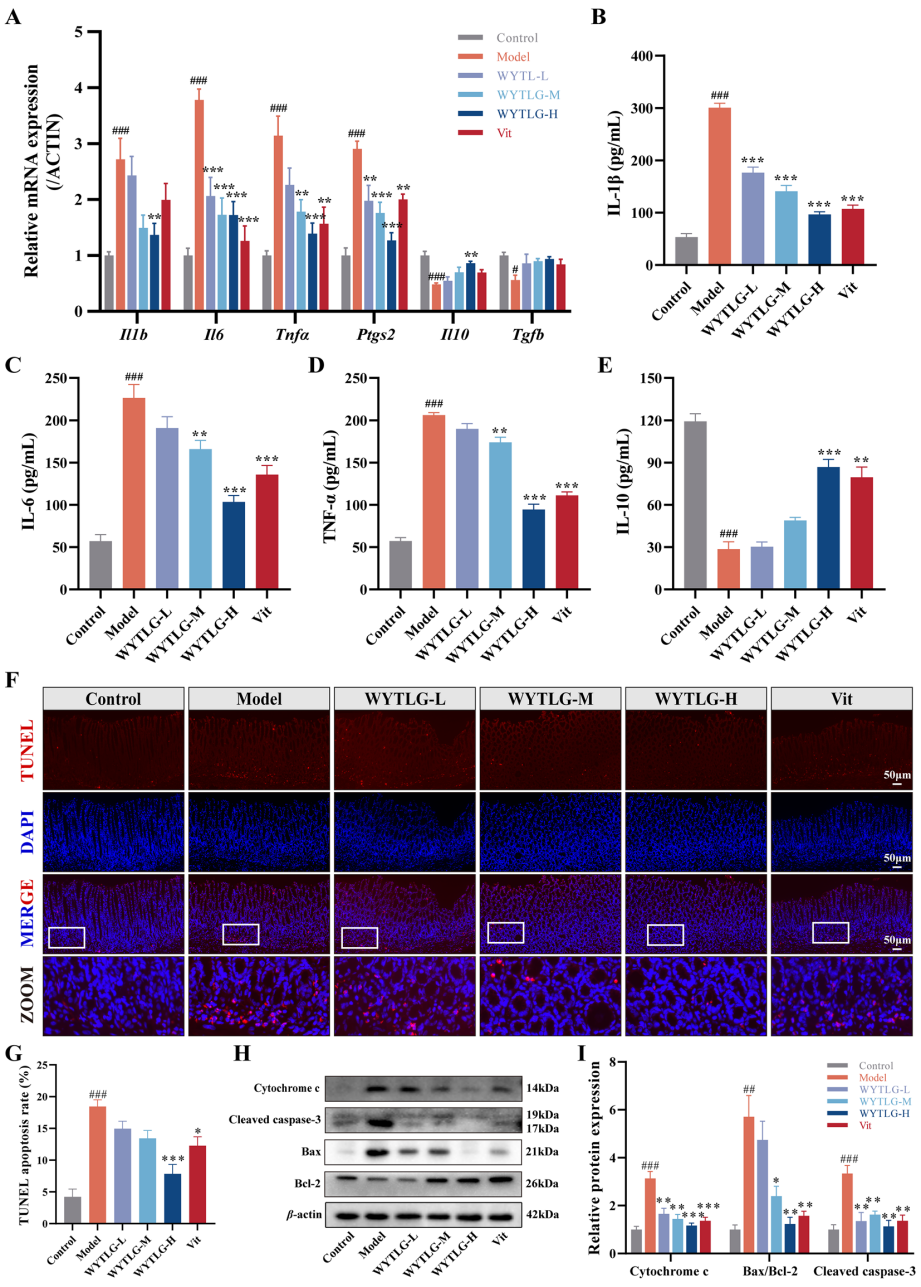
Effects of WYTLG on inflammatory factors and apoptosis in GIM rats. **A** Relative mRNA expression levels of pro-inflammatory cytokines (*Il1b*, *Il6*, *Tnfα*, *Ptgs2*) and anti-inflammatory cytokines (*Il10*, *Tgfb*) in gastric tissues. **B-E** Serum levels of pro-inflammatory (IL-1β, IL-6, TNF-α) and anti-inflammatory (IL-10) cytokines. **F, G** TUNEL staining and semi-quantitative analysis of apoptotic cells (scale bar = 50 μm). **H, I** Western blot analysis of cytochrome c, Bax/Bcl-2, and cleaved caspase-3 protein expression. (*n* = 3–8). ^#^*p* < 0.05, ^##^*p* < 0.01, ^###^*p* < 0.001 *vs.* control group; ^*^p < 0.05, ^**^p < 0.01, ^***^p < 0.001 *vs.* model group

GIM development is closely associated with chronic inflammation and apoptosis dysregulation. WYTLG significantly decreased pro-inflammatory factors (*Il1b*, *Il6*, *Tnfα*, and *Ptgs2*) while increasing anti-inflammatory factors (*Il10* and *Tgfb*) in gastric tissues, with the WYTLG-H group showing the most pronounced effects (*p* < 0.001) (Fig. 4A). Similar trends were noted at the serological level (Fig. 4B-E), indicating that WYTLG mitigates both localized and systemic inflammatory responses.

TUNEL staining revealed elevated apoptosis rates in the GIM model group (*p* < 0.001), which were significantly reduced following WYTLG-H and Vit treatment (*p* < 0.001, *p* < 0.05) (Fig. 4F, G). Furthermore, WYTLG downregulated the protein levels of mitochondrial cytochrome c, apoptotic effector cleaved caspase-3, and the pro-apoptotic factor Bax, while upregulating the anti-apoptotic factor Bcl-2 (Fig. 4H, I). These results demonstrate that WYTLG modulates the balance between apoptosis and anti-apoptosis in GIM rats.

### WYTLG regulates PPARγ/NF-κB signaling pathway in GIM rats

**Fig. 5:**
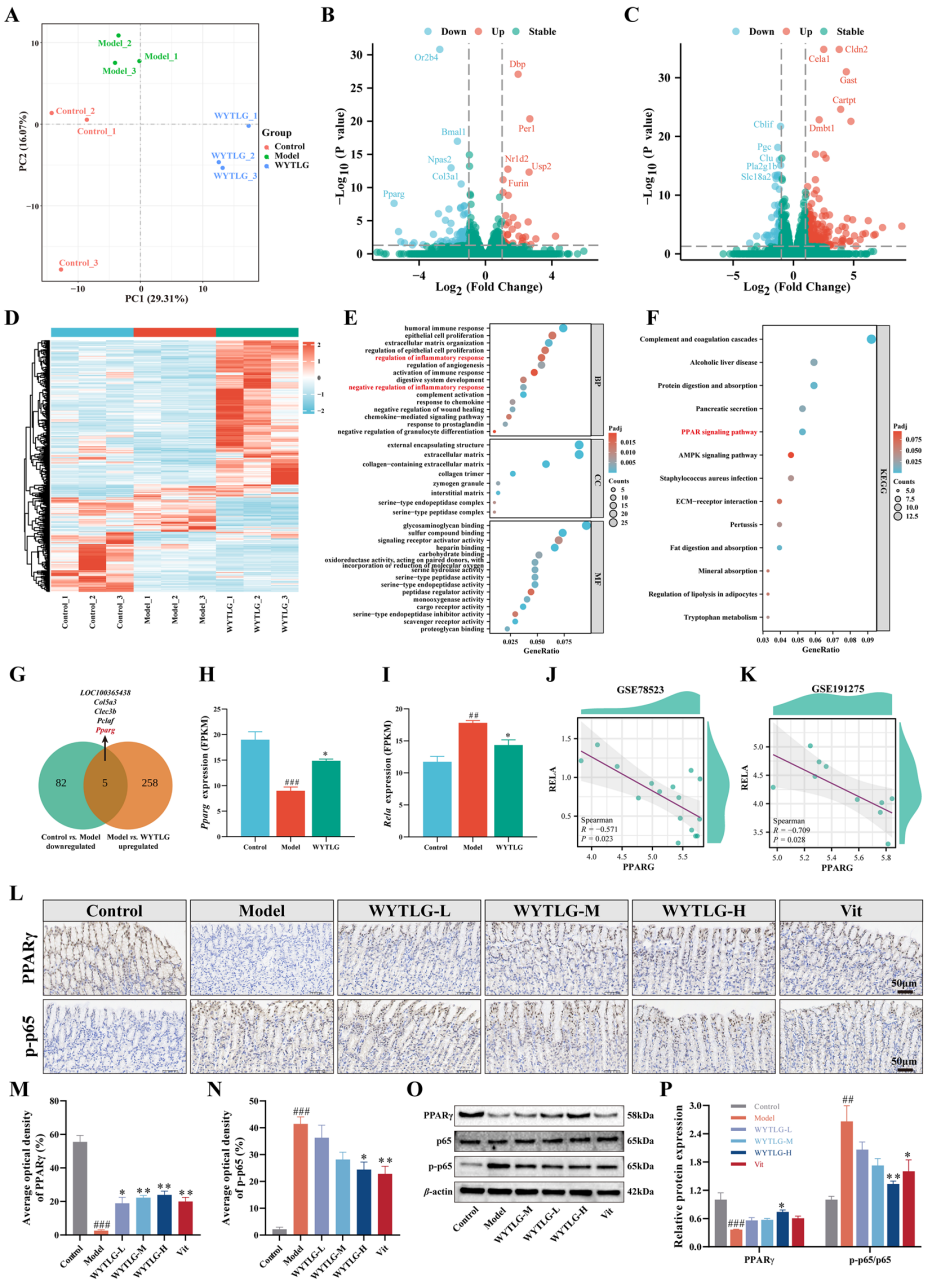
Transcriptomic profiling and validation of PPARγ/NF-κB signaling pathway in WYTLG-treated GIM rats. **A** PCA of transcriptomic profiles. **B** Volcano plots of DEGs (top 5) between control and model groups. **C** Volcano plot of DEGs (top 5) between model and WYTLG-H groups. **D** Hierarchical clustering heatmap of DEGs across experimental groups. **E** GO enrichment analysis. **F** KEGG pathway enrichment analysis. **G** Overlapping genes between downregulated (control vs. model) and upregulated (model vs. WYTLG-H) groups. **H, I** Transcriptional expression levels (FPKM values) of PPARG and RELA across experimental groups. **J, K** Spearman correlation analysis of PPARG (PPARγ) and RELA (p65) expression using human GIM microarray data from the GEO database (GSE78523 and GSE191275). **L-N** IHC staining and quantification of PPARγ and p-p65 expression (scale bar = 50 μm). **O, P** Western blot analysis of PPARγ and p-p65 protein expression. (*n* = 3). ^##^*p* < 0.01, ^###^*p* < 0.001 *vs.* control group; ^*^p < 0.05, ^**^p < 0.01 *vs.* model group

RNA sequencing was performed to explore the potential mechanisms of WYTLG in treating GIM. All samples exhibited good RNA integrity throughout extraction and processing, meeting the quality requirements for high-throughput RNA sequencing (Supplementary Tables S5-6). Principal component analysis (PCA) revealed significant inter-group differences and good intra-group reproducibility (Fig. 5A; Fig. S2). The two principal components collectively explained 45.38% of the total transcriptional variance (PC1: 29.31%; PC2: 16.07%), with PC1 primarily capturing treatment-related variations and PC2 reflecting secondary biological variability. Volcano plot analysis identified 122 DEGs between the control and model groups (35 upregulated, 87 downregulated) and 341 DEGs between the model and WYTLG-H groups (263 upregulated, 78 downregulated) (Fig. 5B, C; Table S7). Heatmap analysis further illustrated distinct expression patterns of DEGs among the three groups (Fig. 5D). GO analysis revealed enrichment of DEGs in inflammation and immunological biological processes (Fig. 5E). KEGG pathway analysis highlighted significant enrichment in inflammation, immune response, metabolism, and digestion pathways, particularly the PPAR signaling pathway (Fig. 5F). Subsequently, we identified five overlapping genes by intersecting genes downregulated in the control *vs.* model comparison with those upregulated in the model *vs.* WYTLG-H comparison, namely *LOC100365438*, *Col5a3*, *Clec3b*, *Pclaf*, and *Pparg* (Fig. 5G). Notably, *Pparg* is a key gene in the PPAR signaling pathway.

Given the significant enrichment of inflammation-related pathways in both GO and KEGG analyses, we explored the potential interaction between the canonical inflammatory NF-κB pathway and the PPAR pathway by examining the transcriptional levels of PPARγ (also known as PPARG) and p65 (also known as RELA) in the RNA sequencing samples. In the GIM model, PPARγ was markedly decreased (*p* < 0.001), whereas RELA was notably increased (*p* < 0.001), and these changes were reversed by WYTLG-H intervention (*p* < 0.05) (Fig. 5H, I). Bioinformatics analysis of publicly available human gastric arrays revealed a significant negative correlation between PPARγ and RELA expression in GIM samples (*p* < 0.05) (Fig. 5J, K). Furthermore, protein-level validation (Fig. 5L-P) confirmed that PPARγ was dose-dependently upregulated, whereas p-p65 was dose-dependently downregulated by WYTLG, in line with the overall trend observed in transcriptomic analysis.

### DCA induces intestinal phenotype in gastric cells

**Fig. 6:**
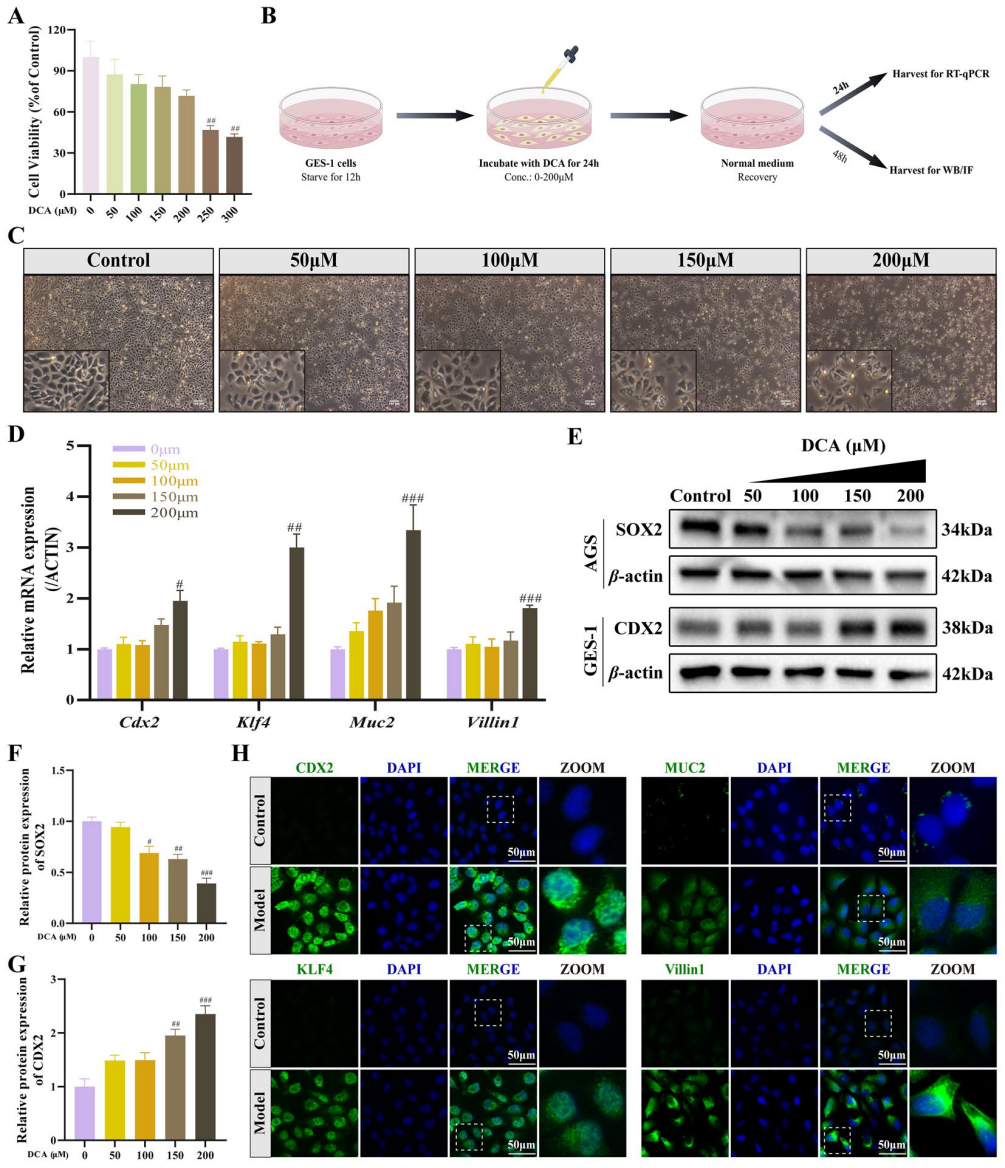
Establishment and validation of an in vitro GIM model induced by DCA. **A** Determination of experimental DCA concentration. **B** Schematic diagram for GIM cell model establishment through DCA stimulation (generated by figdraw.com). **C** Microscopic examination of morphological changes after DCA treatment (scale bar = 100 μm). **D** Relative mRNA expression levels of GIM-specific markers (*Cdx2*, *Klf4*, *Muc2*, and *Villin1*) in DCA-treated GES-1 cells. **E–G** Western blot analysis and quantification of intestinal differentiation marker CDX2 protein expression in GES-1 cells and gastric epithelial marker SOX2 protein expression in AGS cells. **H** Representative IF images of CDX2, KLF4, MUC2, and Villin1 in DCA-treated GES-1 cells (scale bar = 50 μm). (*n* = 3). ^#^*p* < 0.05, ^##^*p* < 0.01, ^###^*p* < 0.001 *vs.* control group

To investigate intestinal metaplasia–like phenotypic changes in vitro, a DCA-induced GIM cell model was established using GES-1 cells (Fig. 6B). CCK-8 assays showed that low concentrations of DCA (50–200 μM) did not significantly affect cell viability compared with the untreated control (Fig. 6A). In contrast, 250 μM DCA reduced cell viability by 53.1% (*p* < 0.01), while 300 μM DCA exhibited enhanced cytotoxicity (*p* < 0.01). Morphological observation revealed that DCA treatment (0–200 μM) resulted in reduced cell adhesion and increased intercellular spacing, indicating that DCA exerts cytotoxic effects at higher concentrations on GES-1 cells (Fig. 6C).

Further analysis demonstrated that DCA dose-dependently increased the mRNA expression of intestinal markers, including *Cdx2*, *Klf4*, *Muc2*, and *Villin1* (*p* < 0.05, *p* < 0.01, *p* < 0.001), with maximal induction observed at 200 μM (Fig. 6D). Previous studies [33] have shown that SOX2, a key regulator of gastric development, is absent in GES-1 cells but highly expressed in GC cells, whereas CDX2, a marker of intestinal differentiation, is progressively upregulated during GIM. Consistently, Western blot analysis revealed increased CDX2 expression in DCA-treated GES-1 cells and reduced SOX2 expression in AGS cells (Fig. 6E-G). Based on these results, 200 μM DCA was selected for subsequent experiments. Immunofluorescence analysis further confirmed that 200 μM DCA treatment for 24 h enhanced nuclear localization of CDX2 and KLF4, cytoplasmic expression of MUC2, and membrane-associated expression of Villin1 in GES-1 cells (Fig. 6H). Together, these results suggest that DCA can induce intestinal phenotype while suppressing gastric phenotype in gastric epithelial cells.

### Therapeutic effects of WYTLG-containing serum on GIM cells

**Fig. 7:**
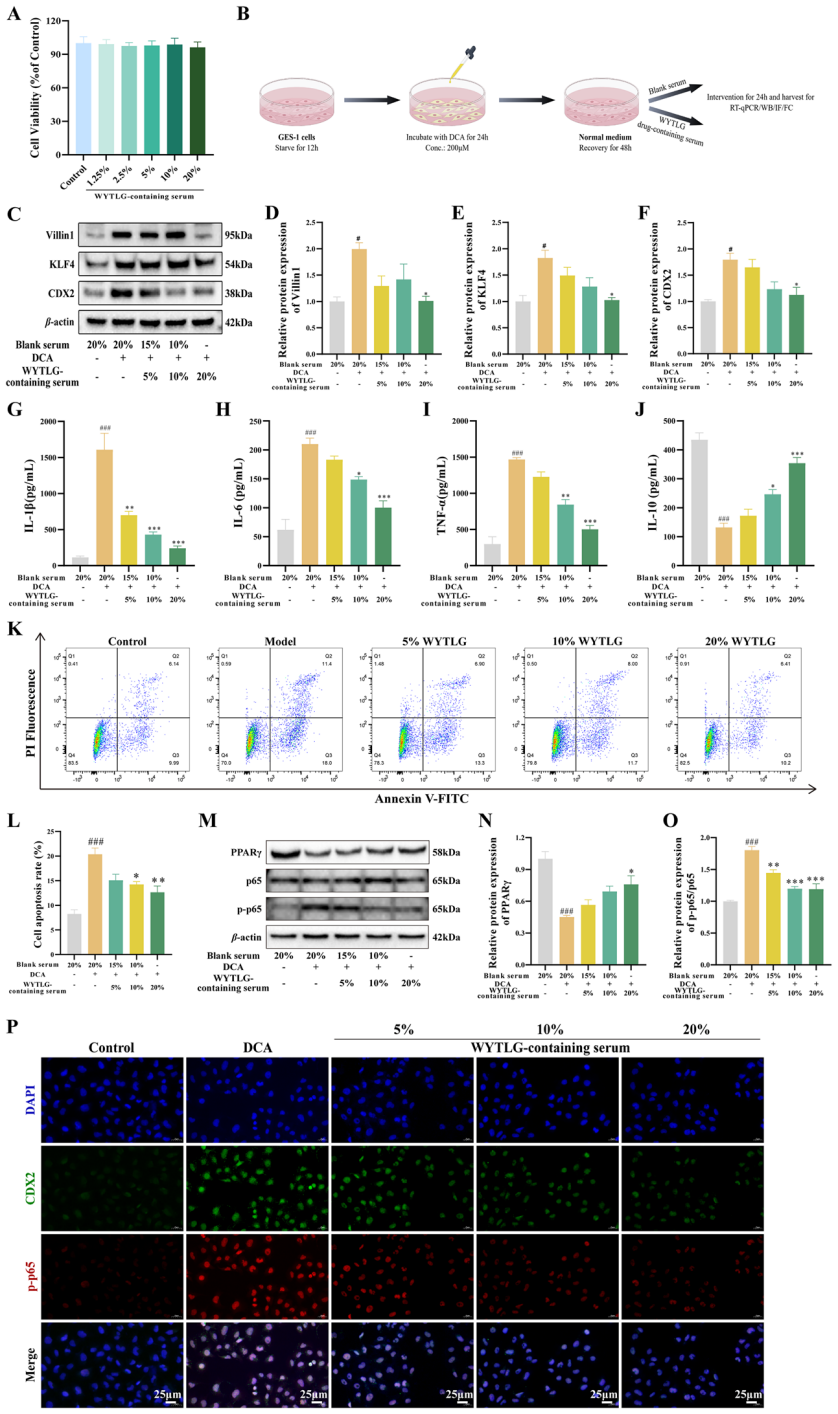
WYTLG-containing serum reverses GIM, inflammation, and apoptosis in DCA-induced GIM cells while modulating PPARγ/NF-κB pathway.** A** Determination of experimental concentration of WYTLG-containing serum. **B** Schematic diagram for drug-containing serum intervention on GIM cells (generated by figdraw.com). **C-F** Western blot analysis and quantification of intestinal phenotype markers (CDX2, KLF4, and Villin1) protein expression after WYTLG treatment. **G-J** Inflammatory cytokine levels in GIM cells following WYTLG treatment. **K, L** Apoptosis rate analysis by flow cytometry. **M–O** Western blot analysis and quantification of PPARγ and p-p65 after WYTLG treatment. **P** IF co-localization of p-p65 and CDX2 in GIM cells after WYTLG treatment (scale bar = 25 μm). (*n* = 3). ^#^*p* < 0.05, ^###^*p* < 0.001 *vs.* control group; ^*^p < 0.05, ^**^p < 0.01, ^***^p < 0.001 *vs.* model group

To investigate the pharmacodynamic effects of WYTLG at the cellular level, WYTLG-containing serum was prepared (Fig. 7B). CCK-8 assays showed that WYTLG-containing serum at various concentrations did not significantly affect the viability of GES-1 cells (Fig. 7A). GIM cells were subsequently treated with 5%, 10%, and 20% WYTLG-containing serum to evaluate the effects on intestinal metaplasia, inflammation, apoptosis, and the expression of pathway-related regulators. As shown in Fig. 7C-F and Fig. S1, WYTLG-containing serum significantly reduced the protein expression of Villin1, KLF4, MUC2, and CDX2. ELISA assays of cell supernatants revealed a concentration-dependent decrease in pro-inflammatory cytokines (IL-1β, IL-6, TNF-α) and an increase in the anti-inflammatory cytokine IL-10 (Fig. 7G-J). Flow cytometry analysis demonstrated that WYTLG-containing serum dose-dependently attenuated apoptosis in GIM cells. (Fig. 7K, L). Additionally, WYTLG-containing serum upregulated PPARγ expression and reduced nuclear p-p65 levels (Fig. 7M-O), consistent with in vivo efficacy. IF co-localization analysis further showed decreased nuclear co-localization of CDX2 and p-p65 with increasing concentrations of WYTLG-containing serum (Fig. 7P). Collectively, these results suggest that WYTLG-containing serum alleviates GIM-related alterations in vitro by modulating intestinal metaplasia, inflammation, and apoptosis, potentially through regulation of the PPARγ/NF-κB signaling pathway.

### WYTLG-containing serum regulates PPARγ/NF-κB signaling pathway in GIM cells

**Fig. 8:**
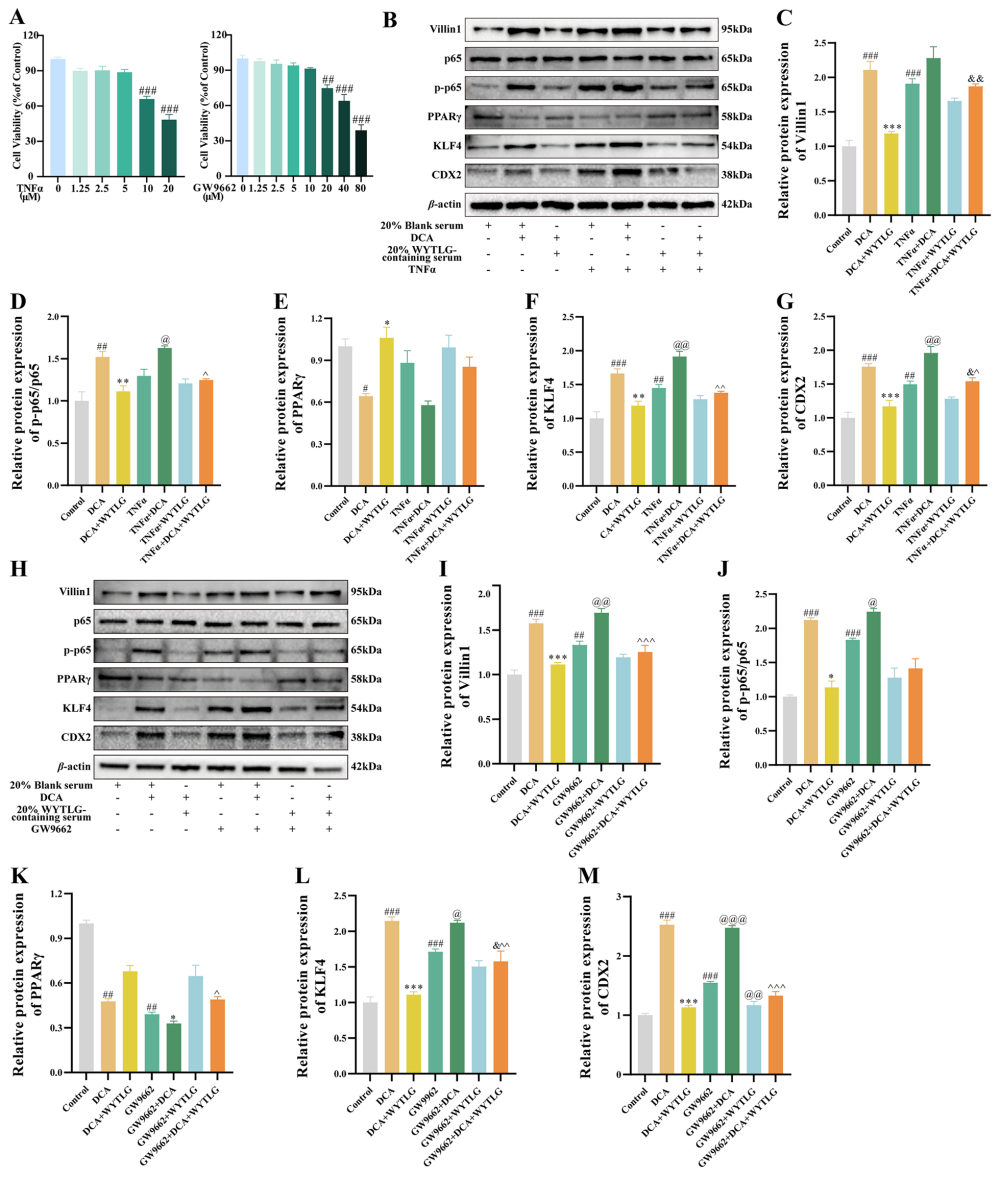
Effects of WYTLG-containing serum on the PPARγ/NF-κB signaling pathway in GIM cells.** A** Determination of experimental TNFα and GW9662 concentrations. **B-G** Western blot analysis and quantification of PPARγ/NF-κB signaling pathway and intestinal phenotype markers in GIM cells treated with TNFα (NF-κB pathway activator). **H-M** Western blot analysis and quantification of PPARγ/NF-κB signaling pathway and intestinal phenotype markers in GIM cells treated with GW9662 (PPARγ antagonist). (*n* = 3). ^#^*p* < 0.05, ^##^*p* < 0.01, ^###^*p* < 0.001 *vs.* control group; ^*^p < 0.05, ^**^p < 0.01, ^***^p < 0.001 *vs.* DCA group; ^&^*p* < 0.05, ^&&^*p* < 0.01 *vs.* DCA + WYTLG group; ^@^*p* < 0.05, ^@@^*p* < 0.01, ^@@@^*p* < 0.001 *vs.* TNFα/GW9662 group; ^^^*p* < 0.05, ^^^^*p* < 0.01, ^^^^^*p* < 0.001 *vs.* TNFα/GW9662 + DCA group

Our previous results have demonstrated WYTLG’s dual regulatory effects on PPARγ upregulation and p-p65 suppression in vivo and in vitro. Considering that p-p65 can interact with CDX2 to regulate downstream intestinal markers (validated by IF experiments), we further employed GW9662 (a PPARγ antagonist) and TNF-α (an NF-κB activator) for mechanistic validation. The optimal concentrations of agents were first determined (Fig. 8A). Subsequently, cells were pre-treated with 5 μM TNF-α or 10 μM GW9662, either alone or in combination with WYTLG.

As shown in Fig. 8B-G, TNF-α significantly enhanced nuclear p-p65 levels (*p* < 0.05), which was partially attenuated by WYTLG (*p* < 0.05). Notably, TNF-α exhibited no significant effect on PPARγ expression (*p* > 0.05) but substantially reversed WYTLG-mediated downregulation of intestinal markers (Villin1, KLF4, and CDX2; *p* < 0.01, *p* < 0.001). These findings suggest that, NF-κB activation predominantly affects downstream signaling, with no significant change in PPARγ expression detected. In addition, as shown in Fig. 8H-M, GW9662 significantly suppressed PPARγ expression (*p* < 0.01) while elevating p-p65 levels, both of which were partially restored by WYTLG treatment. The GW9662-induced upregulation of intestinal markers (*p* < 0.05) was also counteracted by WYTLG, suggesting that PPARγ suppression triggers NF-κB activation and subsequent intestinal marker expression. Taken together, these results demonstrate that WYTLG ameliorates GIM via a PPARγ/NF-κB/CDX2-dependent mechanism.

### WYTLG-containing serum attenuates NF-κB activation and GIM in a PPARγ-dependent manner

**Fig. 9:**
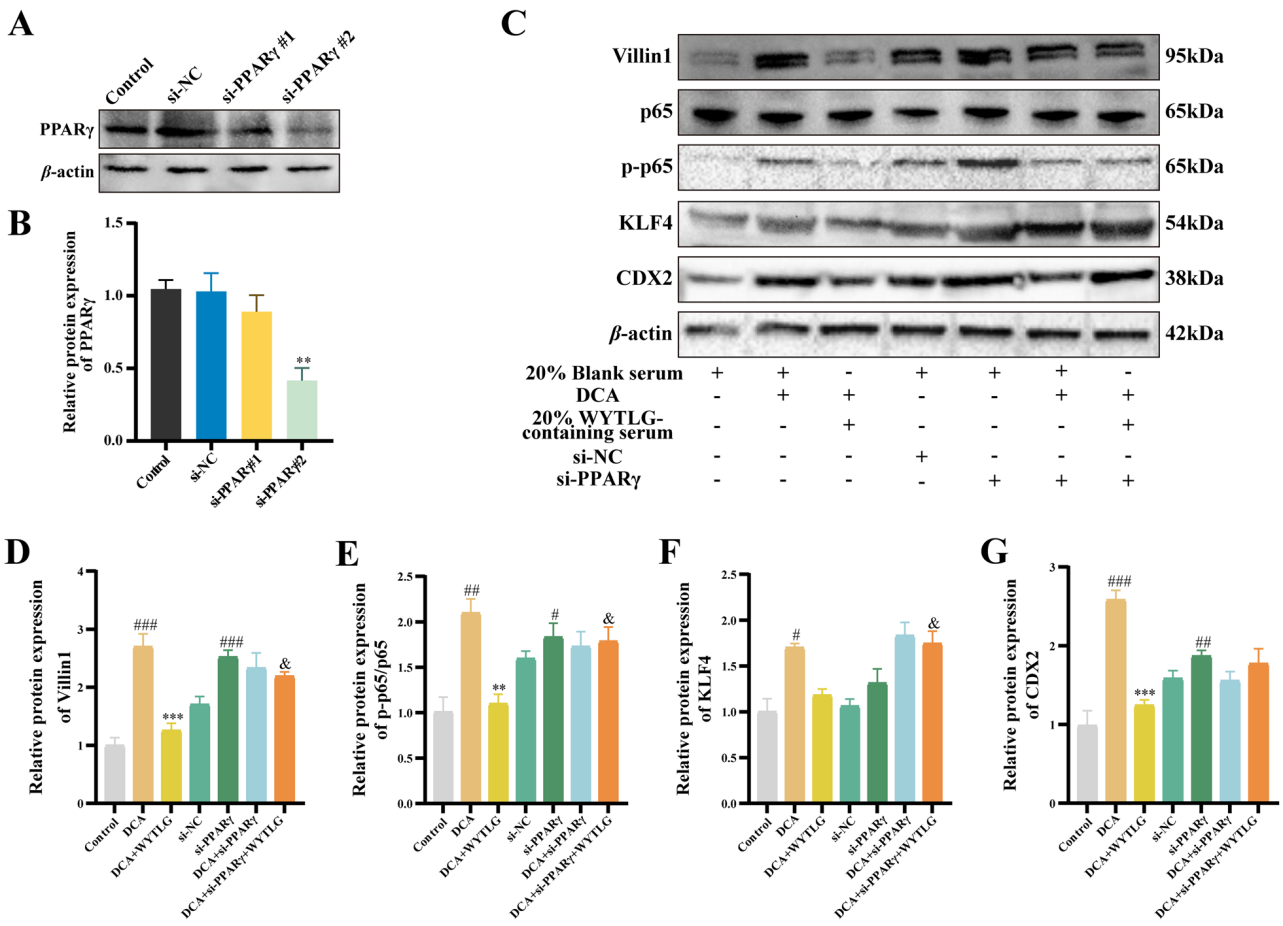
WYTLG-containing serum attenuates NF-κB activation in GIM in a PPARγ-dependent manner. **A, B** Western blot analysis and quantification of PPARγ. **C-G** Western blot analysis and quantification of Villin1, p-p65/p65, KLF4, and CDX2. (*n* = 3). ^#^*p* < 0.05, ^##^*p* < 0.01, ^###^*p* < 0.001 *vs.* control group; ^**^p < 0.01, ^***^p < 0.001 *vs.* DCA group; ^&^*p* < 0.05 *vs.* DCA + WYTLG group

To investigate whether WYTLG suppresses GIM progression by upregulating PPARγ and subsequently inhibiting NF-κB, we transfected GES-1 cells with si-PPARγ to verify this mechanism. As shown in Fig. 9A, B, both si-PPARγ sequences effectively reduced PPARγ protein expression, with si-PPARγ#2 exhibiting better knockdown efficiency and therefore being selected for subsequent experiments. WYTLG weakened the reduction in p-p65 levels and GIM-associated markers, including Villin1, KLF4, and CDX2 in the absence of PPARγ (Fig. 9C-G). These results revealed that the protective effects of WYTLG against NF-κB/CDX2 pathway activation are in a PPARγ-dependent manner, thereby attenuating the progression of GIM.

In addition, molecular docking analysis was performed to explore the potential interactions between active compounds of WYTLG and proteins involved in the PPARγ/NF-κB/CDX2 signaling pathway. The binding energies of the receptor-ligand complexes were ranked and visualized by heatmap clustering, with all values below −1.2 kcal/mol, indicating acceptable docking stability (Fig. S3).

## Discussion

GIM itself may be interpreted as an adaptive and benign response to the adverse gastric milieu. Since the Correa’s cascade was proposed, the concept of GIM as a true precursor of GC, especially of the intestinal-type has been widely accepted [23]. Despite advances in understanding the pathogenesis of GIM, effective treatments remain limited. In recent years, complementary and alternative medicine has gained widespread attention for its unique efficacy in managing GPL. Among these, WYTLG has shown promise in alleviating GIM symptoms, although the mechanisms of action are not fully understood. Here, we systematically evaluated the therapeutic effects of WYTLG on GIM and explored its potential molecular mechanisms using integrated in vivo, in vitro, and in silico approaches.

To achieve standardization, establishing rigorous quality control measures for WYTLG is critical for understanding its mechanisms of action. Among the twenty-nine compounds identified through UPLC-MS/MS, 9 were derived from *Astragali radix* (Huangqi in Chinese), a time-honored tonic herbal medicine known for its “invigorating the spleen and replenishing Qi” efficacy. This pharmacological action directly aligns with the etiology of GIM as a syndrome of Qi deficiency in spleen and stomach [6, 24]. Furthermore, *Astragali Radix* serves as the monarch drug (Jun Yao) within the WYTLG formula framework, embodying the primary principle of TCM combinatorial therapy by neutralizing primary disease manifestations through its dominant therapeutic activity.

We successfully established a GIM animal model by administering MNNG and DCA combined with irregular fasting regimens, simulating detrimental human behaviors such as habitually excessive nitrite consumption, bile reflux, and irregular dietary patterns. The GIM rats exhibited significant physiological impairments, including weight loss, gastric mucosal injury, abnormal serum pepsinogen levels, and histopathological changes indicative of gastric damage. Remarkably, WYTLG intervention led to notable improvements in the overall condition of the rats. Serological markers including pepsinogen and gastrin levels were restored, indicating a recovery of the progressive hypochlorhydria due to glandular atrophy [13, 25]. Notably, gold-standard histopathological assessments revealed a reduction in goblet cells, characteristic of metaplasia [25], demonstrating that WYTLG effectively reversed the pathological alterations associated with GIM.

Clinical evidence indicates that bile acids (BAs) in the stomach are strongly associated with an increased risk of GIM and GC, irrespective of Helicobacter pylori infection [14, 26, 27]. DCA, one of the most hydrophobic and cytotoxic secondary BAs in humans, is a major component of duodenogastric reflux [28]. Therefore, we chose DCA as the inducer for constructing in vivo and in vitro GIM models. In the stomach, CDX2 serves as a key driver of intestinal differentiation [28, 29] and a leading transcription factor (TF) regulating GIM and Barrett’s metaplasia [30, 31]. Furthermore, CDX2 facilitates the transdifferentiation of GIM by promoting the transcription of intestinal markers such as Villin1, KLF4, and MUC2 [27, 30]. In Barrett’s metaplasia, bile reflux enhances CDX2 expression via the NF-κB signaling pathway [32]. In contrast, SOX2 is a critical TF for gastric and esophageal differentiation [33]. Studies have demonstrated an inverse correlation between SOX2 and CDX2 expression in GIM tissues [34, 35]. In our study, WYTLG significantly attenuated the aberrant expression of metaplasia-related TFs while upregulating SOX2 at both mRNA and protein levels. This finding reveals the role of WYTLG in promoting the maintenance of the gastric phenotype by inhibiting metaplasia-related pathways, thereby offering new insights into the treatment of GIM.

Perpetuation of chronic inflammation is a critical driver of the malignant transformation of GIM [26]. While mild inflammation typically maintains a stable state, severe inflammatory insults (e.g., advanced atrophy and hypochlorhydria) or exposure to cytotoxic agents (e.g., nitrosamines and bile acids) trigger the inflammatory response potentially leading to dysplasia or neoplasia and propagating a vicious cycle [23]. In this context, pro-inflammatory mediators such as IL-1β, IL-6, TNF-α, and PTGS2 ​orchestrate gastric epithelial remodeling and activate the pro-apoptotic gene Bax, thereby disrupting the balance of apoptosis [9, 36]. Normally, the balance between pro-apoptotic proteins like Bax and anti-apoptotic proteins like Bcl-2 is crucial in determining cell fate. When cellular stress or damage occurs, Bax translocates to the mitochondria, inducing changes in mitochondrial membrane permeability. The disruption of the mitochondrial membrane by Bax facilitates the release of cytochrome c from the intermembrane space into the cytosol, leading to the formation of the apoptosome complex and subsequent activation downstream effector proteins, including cleaved-caspase-3, which carry out the cellular dismantling required for programmed cell death [37–39]. WYTLG not only significantly attenuated the production of inflammatory factors both in vivo and in vitro but also enhanced the expression of anti-inflammatory factors. By regulating the mitochondrial pathway, WYTLG inhibited the release of pro-apoptotic proteins, thereby effectively protecting cells from apoptotic damage.

Through integrated transcriptomic and bioinformatics analysis, PPARγ was identified as the key functional target of WYTLG in gastric tissue. Among the PPAR isoforms, PPARγ represents the most extensively characterized subtype in the gastrointestinal tract and the sole isoform conserved across human and murine system [40]. PPARγ agonists have been shown to promote gastric acid secretion and accelerate ulcer healing by upregulating serum and glucocorticoid-induced kinase (SGK1) [41]. Furthermore, PPARγ exhibits functional dysregulation in various malignancies including GC. PPARγ heterozygous-deficient mice exhibit increased susceptibility to carcinogen-induced GC and reduced survival rates [42]. However, the relationship between PPARγ and GPL remains elusive. Recent studies have revealed that PPARγ interacts with NF-κB through a transrepression mechanism, inhibiting the activation of its target genes, particularly the p65 subunit, in a ligand-dependent manner [40]. This process is independent of PPAR response elements binding and instead involves the recruitment and stabilization of corepressor complexes on pro-inflammatory gene promoters, which may partially account for PPARγ’s anti-inflammatory properties [43]. Notably, CDX2 upregulation is considered a result of chronic gastritis and sustained NF-κB activation [44, 45]. Excessive NF-κB activation is recognized as a major cause of pro-inflammatory cytokine release that may contribute to cancer promotion [45]. Our previous studies have also identified NF-κB as a critical regulatory pathway for the activation of intestinal transcription factors [12]. Based on these observations, we propose that PPARγ modulates CDX2 expression by regulating the NF-κB pathway. Our research provides evidence that NF-κB can colocalize with CDX2 in the nucleus, and its fluorescence intensity significantly decreases after WYTLG intervention and these findings are consistent with existing literature [45]. Importantly, the present study extends our earlier observations by identifying PPARγ as a potential metabolic and transcriptional checkpoint linking bile acid-induced stress to NF-κB activation and CDX2-driven intestinal differentiation.

**Fig. 10:**
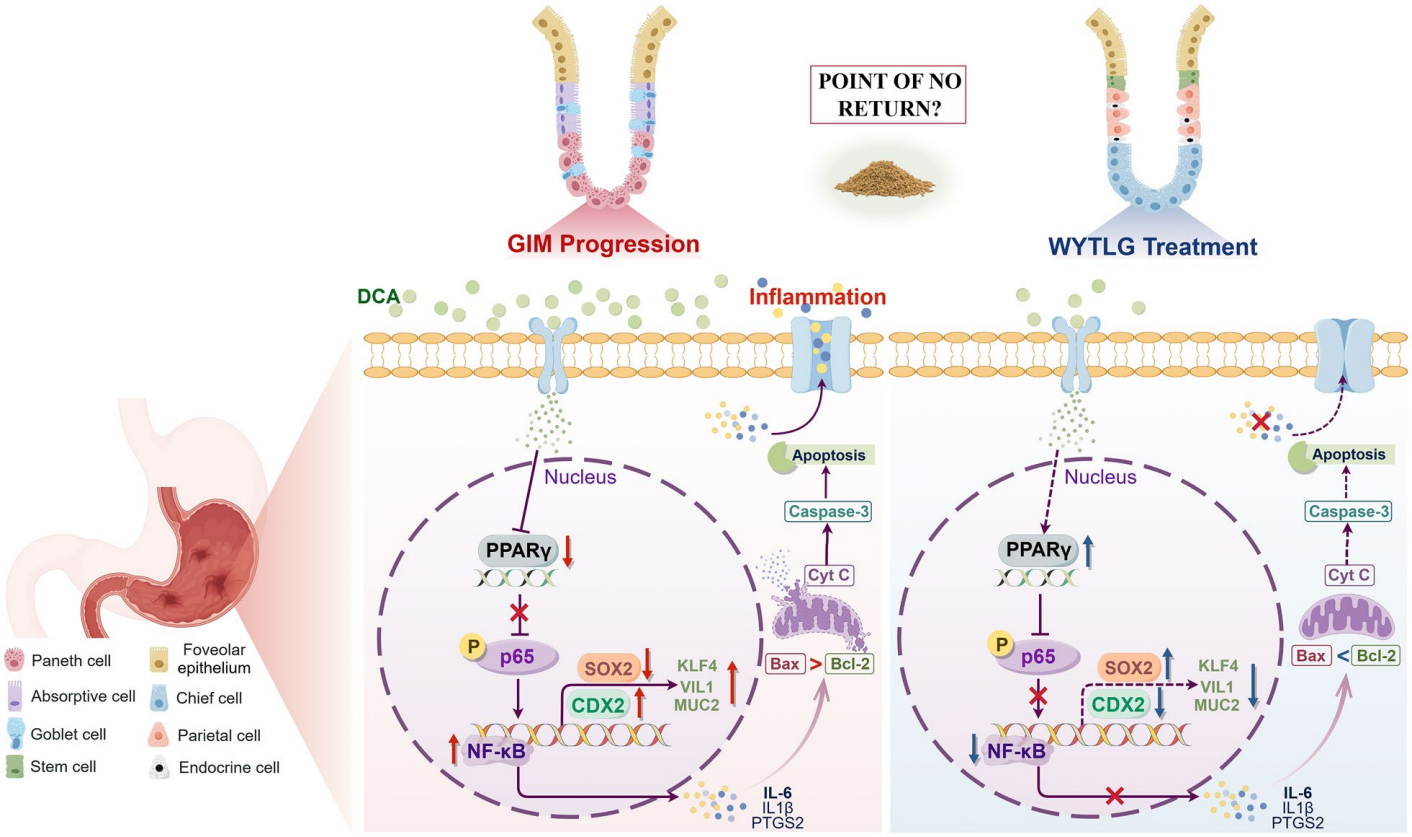
Schematic illustration of the mechanism by which WYTLG regulated PPARγ/NF-κB/CDX2 pathway in the treatment of GIM. Bile acid reflux induces PPARγ downregulation, triggering NF-κB-mediated inflammation via p-p65 nuclear translocation. This cascade promotes CDX2-driven intestinal differentiation (KLF4/Villin1/MUC2) and suppresses gastric SOX2 while activating apoptotic pathways

In summary, we have deciphered the molecular mechanism of WYTLG against GIM (Fig. 10). As depicted, in gastric epithelial cells, DCA leads to the downregulation of PPARγ, resulting in sustained activation of the NF-κB pathway and nuclear translocation of p-p65, which subsequently amplifies chemokine secretion and promotes CDX2 transcription and intestinal differentiation. These findings suggest that PPARγ may function as a key transcriptional regulator coordinating the phenotypic evolution and progression of GIM. The newly identified WYTLG mediated PPARγ/NF-κB/CDX2 axis provides novel insights into the regulatory mechanisms of CDX2 in GIM, offering potential avenues for the early detection and prevention of malignant transformation. Nevertheless, future studies should prioritize ​elucidating the direct mechanistic interplay​ between WYTLG-mediated PPARγ activation and NF-κB signaling axis modulation, which will provide a more comprehensive rationale for the potential use of WYTLG in the treatment of GIM.

## Conclusion

This study reveals that WYTLG ​ameliorates gastric mucosal inflammation, apoptosis, and intestinal metaplasia by modulating the PPARγ/NF-κB/CDX2 axis, offering novel insights into gastric cell transdifferentiation and providing ​scientific substantiation for clinical translation​ of WYTLG in GIM management. Nevertheless, there still has limitations. First, WYTLG is a multi-component formula, and the specific active components responsible for its regulatory effects on GIM remain to be fully elucidated. Second, although the present findings provide mechanistic evidence for the therapeutic effects of WYTLG, further validation in clinical cohorts is warranted.

## Supplementary Information

Additional file 1

Additional file 2

Additional file 3

Additional file 4

Additional file 5

## Data Availability

The data associated with this study can be obtained from the corresponding author upon reasonable request.

## Abbreviations

GIM: Gastric intestinal metaplasia
WYTLG: Weiyan Tongluo Granules
TCM: Traditional Chinese medicine
GC: Gastric cancer
GPL: Gastric precancerous lesions
UHPLC: Ultra-high-performance liquid chromatography
MNNG: N-Methyl-N’-nitro-N-nitrosoguanidine
DCA: Deoxycholic acid
Vit: Vitacoenzyme
PFA: Paraformaldehyde
H&E: Hematoxylin and eosin
AB/PAS: Alcian blue/periodic acid-Schiff
ELISA: Enzyme-linked immunosorbent assay
PG Ⅰ: Pepsinogen Ⅰ
PG Ⅱ: Pepsinogen Ⅱ
GAS-17: Gastrin-17
PGR: Pepsinogen ratio
RT-qPCR: Reverse transcription quantitative polymerase chain reaction
IHC: Immunohistochemistry
RT: Room temperature
TUNEL: TdT-mediated dUTP nick-end labeling
FPKM: Transcript per million mapped reads
DEGs: Differentially expressed genes
GEO: Gene Expression Omnibus
IF: Immunofluorescence
CCK-8: Cell counting kit-8
TIC: Total ion chromatograms
PCA: Principal component analysis
BAs: Bile acids
TF: Transcription factor
