# Supplementary material for: Weiyan Tongluo Granules attenuate gastric intestinal metaplasia through PPARγ/NF-κB/CDX2 signaling pathway
Source: Chin Med. 2026 Mar 4;21:75. doi: 10.1186/s13020-026-01350-y (PMC12958734; doi:10.1186/s13020-026-01350-y)
Supplement: Supplementary file 1 — Additional file 1 [file 13020_2026_1350_MOESM1_ESM.docx]

**Fig. S1. WYTLG-containing serum dose-dependently reverses GIM in DCA-induced GIM cells.** Representative IF images of intestinal phenotype markers (CDX2, MUC2, KLF4, and Villin1) (scale bar = 50 μm). (*n* = 3).

**Fig. S2. Transcriptomic data quality assessment and sample relationship analysis.​** (A) Gene expression distribution across all samples. (B) Inter-sample correlation analysis.

**Fig. S3.** **Binding affinities between main chemical components of WYTLG and core pathway regulators.**
