# Supplementary figures and images for: Weiyan Tongluo Granules attenuate gastric intestinal metaplasia through PPARγ/NF-κB/CDX2 signaling pathway
Source: Chin Med. 2026 Mar 4;21:75. doi: 10.1186/s13020-026-01350-y (PMC12958734; doi:10.1186/s13020-026-01350-y)

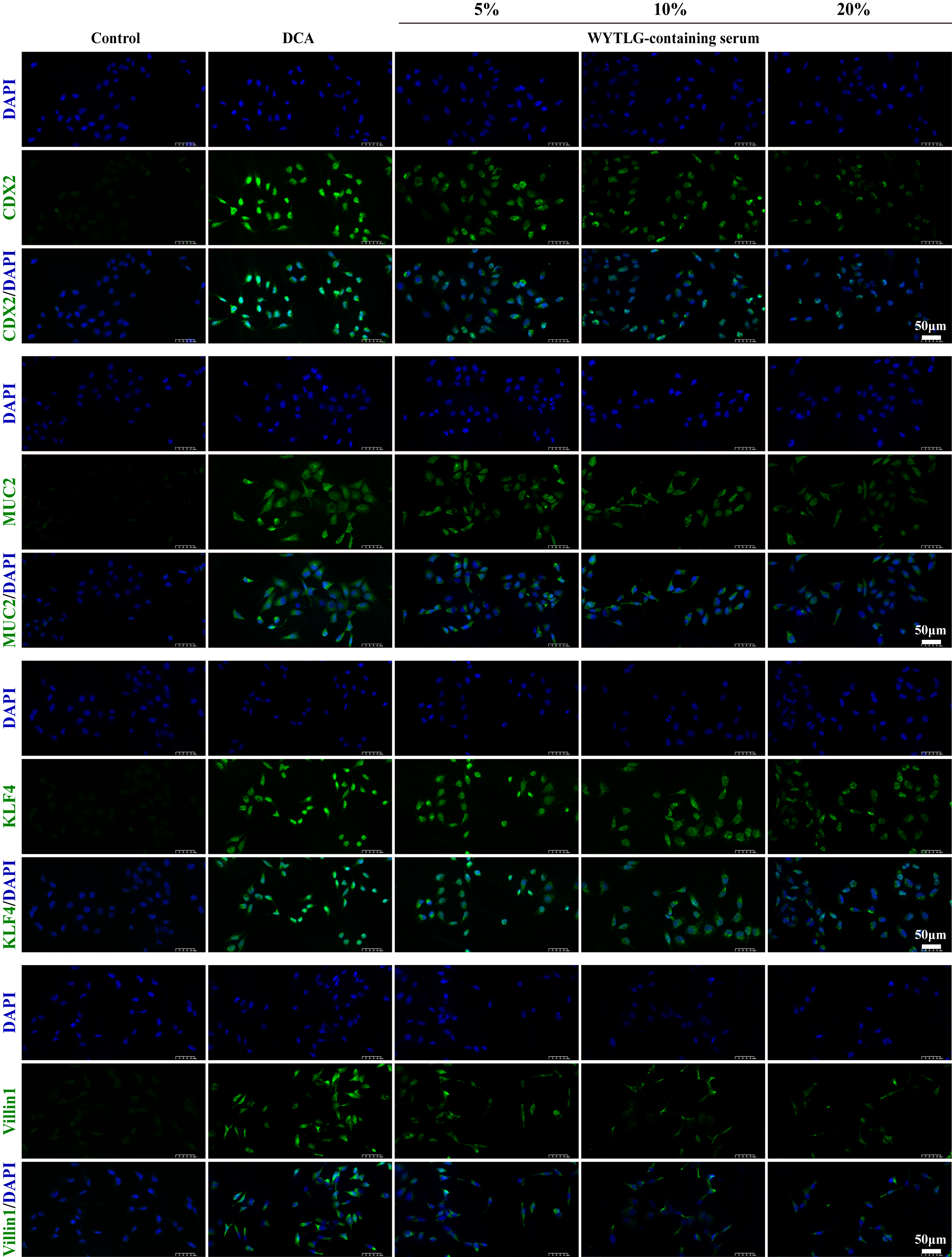

Supplement: Supplementary file 2 — Additional file 2 [file 13020_2026_1350_MOESM2_ESM.tif]

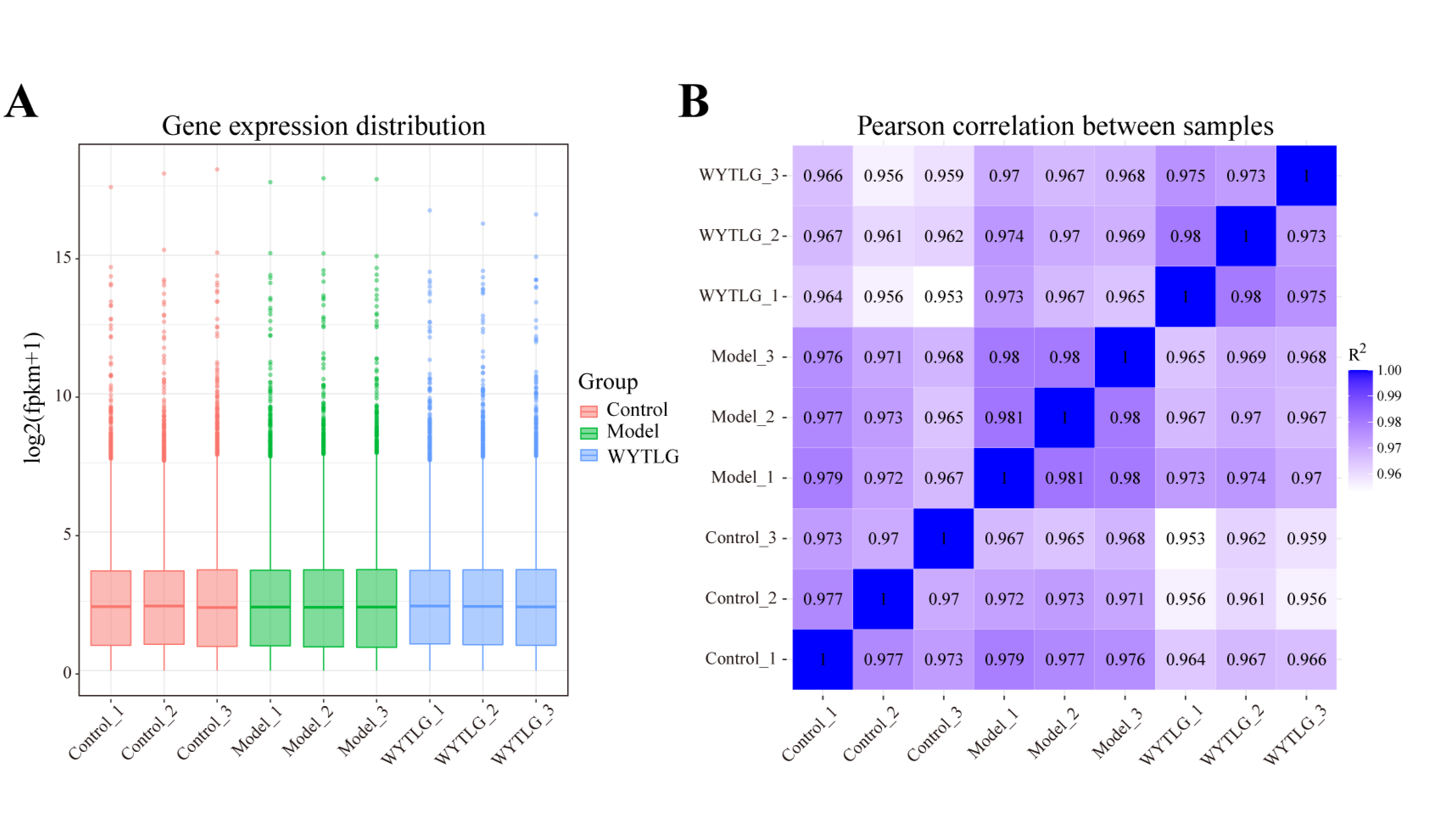

Supplement: Supplementary file 3 — Additional file 3 [file 13020_2026_1350_MOESM3_ESM.tif]

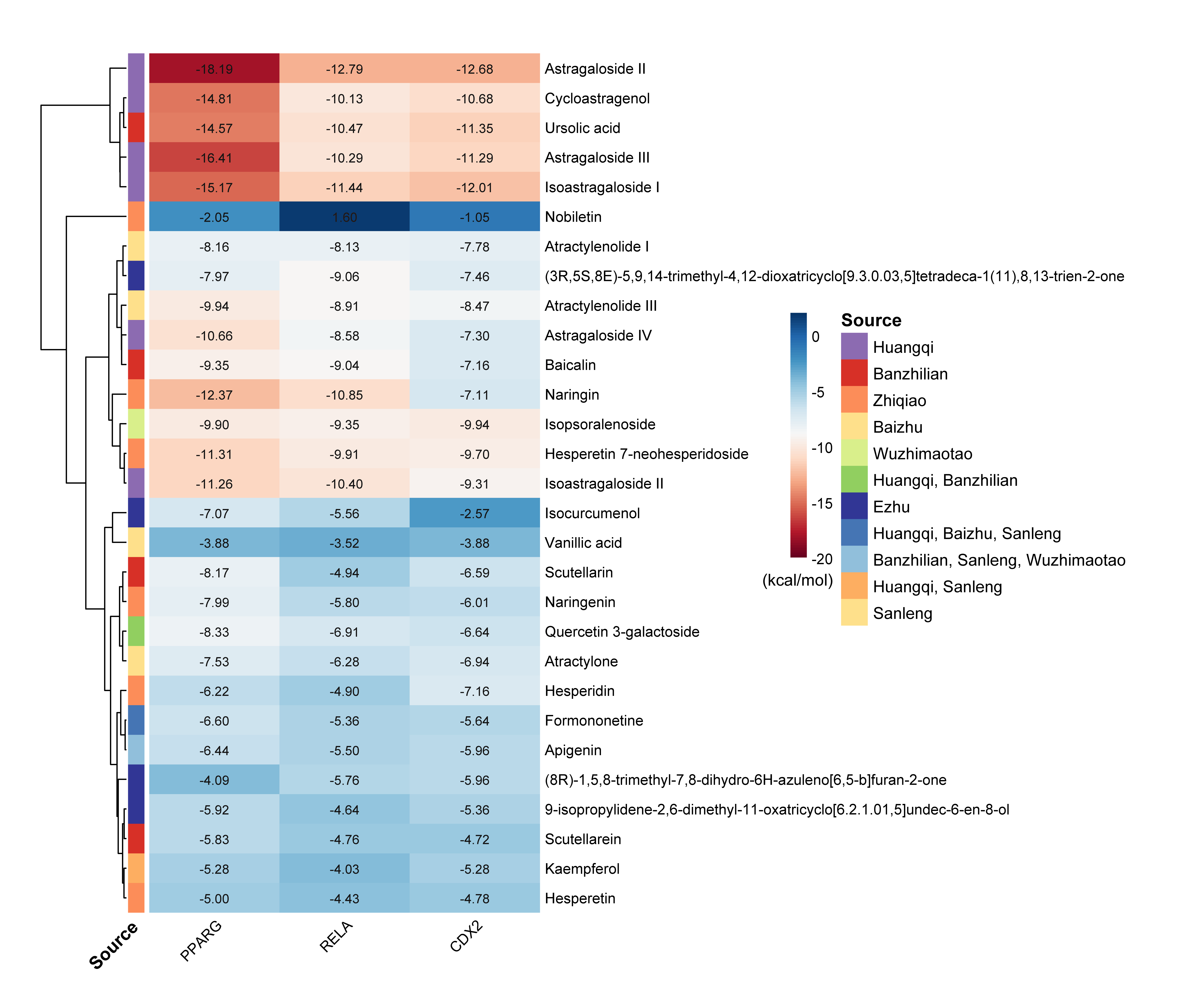

Supplement: Supplementary file 4 — Additional file 4 [file 13020_2026_1350_MOESM4_ESM.tif]
